# Dom34 Links Translation to Protein *O*-mannosylation

**DOI:** 10.1371/journal.pgen.1006395

**Published:** 2016-10-21

**Authors:** Lasse van Wijlick, René Geissen, Jessica S. Hilbig, Quentin Lagadec, Pilar D. Cantero, Eugen Pfeifer, Mateusz Juchimiuk, Sven Kluge, Stephan Wickert, Paula Alepuz, Joachim F. Ernst

**Affiliations:** 1 Department Biologie, Molekulare Mykologie, Heinrich-Heine-Universität, Düsseldorf, Germany; 2 Manchot Graduate School Molecules of Infection, Heinrich-Heine-Universität, Düsseldorf, Germany; 3 Departamento de Bioquímica y Biología Molecular, Universitat de València, Burjassot Spain; 4 ERI Biotecmed. Universitat de València, Burjassot Spain; University of Texas Health Science Center at San Antonio, UNITED STATES

## Abstract

In eukaryotes, Dom34 upregulates translation by securing levels of activatable ribosomal subunits. We found that in the yeast *Saccharomyces cerevisiae* and the human fungal pathogen *Candida albicans*, Dom34 interacts genetically with Pmt1, a major isoform of protein *O-*mannosyltransferase. In *C*. *albicans*, lack of Dom34 exacerbated defective phenotypes of *pmt1* mutants, while they were ameliorated by Dom34 overproduction that enhanced Pmt1 protein but not *PMT1* transcript levels. Translational effects of Dom34 required the 5′-UTR of the *PMT1* transcript, which bound recombinant Dom34 directly at a CA/AC-rich sequence and regulated *in vitro* translation. Polysomal profiling revealed that Dom34 stimulates general translation moderately, but that it is especially required for translation of transcripts encoding Pmt isoforms 1, 4 and 6. Because defective protein *N-* or *O-*glycosylation upregulates transcription of *PMT* genes, it appears that Dom34-mediated specific translational upregulation of the *PMT* transcripts optimizes cellular responses to glycostress. Its translational function as an RNA binding protein acting at the 5′-UTR of specific transcripts adds another facet to the known ribosome-releasing functions of Dom34 at the 3′-UTR of transcripts.

## Author Summary

Fungi respond to damages of their glycostructures in their cell wall by transcriptional upregulation of genes that specify compensatory activities. Upon block of protein *N-*glycosylation, the human fungal pathogen *Candida albicans* increases transcription of *PMT1* encoding a major isoform of protein *O-*mannosyltransferase. Here we demonstrate that the Dom34 protein aids in glycostress responses by upregulating the translation of several *PMT* isoform transcripts. Dom34 has previously been implicated in mechanisms to secure high levels of ribosomal subunits that promote translation in general, e. g. by no-go decay at the 3′-UTR of transcripts. By binding to the 5′-UTR and activating translational initiation of *PMT* transcripts we add a novel mode of action and suggest a preferred class of targets for the translational activities of the Dom34 protein. The combination of transcriptional and Dom34-mediated translational upregulation of *PMT* genes optimizes effective recovery and survival of fungal cells upon glycostress.

## Introduction

In eukaryotes, secretory proteins can get *O*-mannosylated at serine or threonine residues by protein mannosyltransferases (Pmt proteins). This modification occurs during or shortly after translation, during transit across the secretory pore complex into the ER lumen. *O*-mannosylation initiates the typical type of fungal *O*-chains, which mature in the Golgi. In mammalian cells *O*-mannosylation is a rare but important process, while the bulk of *O*-chains is formed post-translationally in the Golgi [1,2]. Seven and five Pmt isoforms forming Pmt1, 2 and 4 subfamilies have been described in the yeast *Saccharomyces cerevisiae* and the human fungal pathogen *Candida albicans*, respectively [1–3]. Pmt isoforms are largely specific for their protein substrates and the lack of the Pmt2 isoform in *C*. *albicans* or at least two isoforms in *S*. *cerevisiae* prevents growth [3,4]. In *C*. *albicans*, each Pmt isoform affects one or another aspect of fungal growth, morphogenesis and virulence [3,5]. Impaired *O*-mannosylation in *pmt1* mutants or upon Pmt1 inhibition of a wild-type strain leads to transcriptional upregulation of *PMT2* and *PMT4* genes, while inhibition of *N-*glycosylation by tunicamycin upregulates *PMT1* expression [6–8]. In both glycostress conditions, the increased levels of underglycosylated proteins also trigger the unfolded protein response (UPR), e. g. by increasing levels of the Kar2 chaperon and matured *HAC1* transcript [6,7]. UPR induction is known to lower overall translation in yeast cells, although translation of some transcripts is increased [9].

In *S*. *cerevisiae*, the Dom34 protein is involved in the “no-go decay” (NGD) process, which is one of at least three mechanisms responding to the quality of translated mRNA. NGD relieves stalled translational complexes arising e. g. by secondary structures or rare codons through dissociation of ribosomal subunits and cleavage of mRNA [10,11]. Dom34 also rescues ribosomes that accumulate at the 3′-UTR of transcripts [12,13]. To promote subunit dissociation and peptidyl-tRNA drop-off from stalled ribosomes, Dom34 co-operates with its associated GTPase Hbs1 [10,11]. Very likely, this function of Dom34 is possible because of its high homology to the translational termination factor eRF1, as well as the structural similarities of the Dom34-Hbs1 and eRF1:eRF3 complexes that can occupy the ribosomal A-site. *S*. *cerevisiae* strains carrying single mutations for Dom34 or Hbs1 grow normally, while their combination with mutations impairing components of 40S (but not 60S) ribosomal subunits [14], mutations delaying translation by phosphorylation of eIF2 [15] and yet undefined mutations [16], leads to severe impairment of growth. The scarcity of 40S subunits due to “stuck” 80S ribosomes and the resultant impaired translational initiation has been suggested as the mechanism causing slow growth of *dom34 hbs1* double mutants [14]. The homology of Dom34 to eRF1 only regards its central and C-terminal domains, while its N-terminal domain adopts a Sm-fold that is characteristic of RNA degradation or recognition domains [17,18]. However, recent results indicated that Dom34 is not the endonuclease that degrades mRNA in stalled ribosomes [19]. Besides NGD, other mechanisms including separation of free 80S ribosomes [14,20] and nonfunctional rRNA decay (NRD) [21,22] depend on Dom34 to maintain a sufficient supply of ribosomes for translation.

Based on the previous discovery of a *S*. *cerevisiae* mutant with defective protein *O*-mannosylation [23] we report here a novel function of Dom34 in translational upregulation of the *PMT1* transcript in *C*. *albicans*. By promoting the translational initiation of the *PMT1* transcript, which under glycostress is strongly increased [6–8], Dom34 contributes to optimize the overall output of Pmt1 activity that helps to recover from damage to its glycostructures. Its mode of action as an RNA binding protein for specific transcripts differs from the previously described general roles and mechanisms of Dom34 in promoting translation in eukaryotes. By this action, Dom34 functionally links two essential processes in eukaryotic cells, translation and *O*-mannosylation.

## Results

### Identification of Dom34 as contributor of Pmt1 function in *S. cerevisiae*

**Fig 1:**
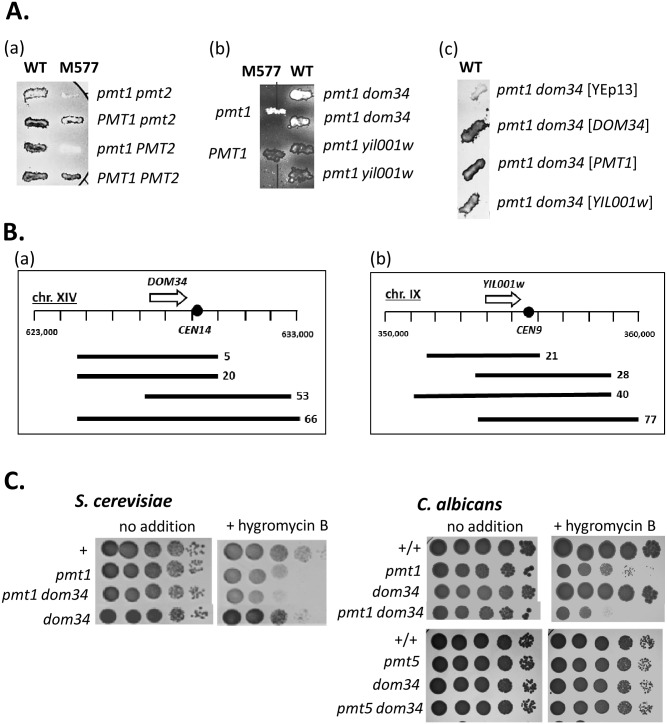
Genetic interactions of *dom34* and *pmt1* mutations. **(A)** Killer phenotypes of *S*. *cerevisiae* mutants. Strains were grown as streaks and replica-printed onto plates containing the killer K1-secreting strain RC130. Following incubation at 18°C for 4–7 d, the appearance of streaks was blue (dead cells stained by methylene blue appear dark on figure), or white (live cells). Strains tested were the parental strain YE449 (WT) and its mutant M577 and *pmt1* and/or *pmt2* derivatives; in addition, mutant strains W21 (*pmt1 dom34*), W12 (*pmt1 yil001w*) and transformants of strain W21 carrying pSW20 (*PMT1*), p577/20 (*DOM34*) and pSW577/2 (*YIL001w*) were tested. **(B)** Inserts in genomic clones complementing the killer K1-resistance of strain M577 *pmt1*. Regions of chromosomes XIV and IX are shown, along with genomic inserts in YEp13 (numbered bars). **(C)** Hygromycin B sensitivity. Strains were serially diluted and spotted on YPD plates without or with hygromycin B (50 μg/ml). Growth was for 2 d at 30°C. *S*. *cerevisiae* strains tested were YE449 (WT), YE449 *pmt1*, YE449 *dom34* and W21 (*pmt1 dom34*). *C*. *albicans* strains tested were CAF2-1 (WT), SPCa2 (*pmt1*/*pmt1*), JH47-1 (*dom34*/*dom34*), JH24-4 (*pmt1*/*pmt1 dom34*/*dom34*), SPCa10 (*pmt5*/*pmt5*) and JH5-3-1 (*dom34*/*dom34 pmt5*/*pmt5*).

Previously, a *S*. *cerevisiae* mutant (M577) defective in *O-*mannosylation of a heterologous protein (hIGF-1) and some homologous secretory proteins had been identified [23]. The mannosylation defect was recessive and segregated 2:2 in crosses to a wild-type strain suggesting that it was caused by mutation of a single gene. Because M577 did not show easily scorable phenotypes, *pmt1* or *pmt2* mutations were introduced to explore synthetic phenotypes with mutations affecting *O-*mannosylation. This approach was led by the finding that *pmt1 pmt2* double mutants, but not the single mutants, are resistant to the K1 killer toxin [24]. In agreement, we found that Pmt^+^- strains YE449 and mutant M577, as well as the *pmt1* and *pmt2* single mutants were toxin-sensitive (blue/dark appearance of colonies on indicator plates), while the *pmt1 pmt2* double mutant was completely resistant to the toxin (white appearance of colonies on indicator plates) (Fig 1Aa). Importantly, similar to the *pmt1 pmt2* double mutant, the *pmt1* derivative of mutant M577 but not of the parental strain YE449 was completely toxin-resistant. Because the *pmt2* derivative of M577 retained sensitivity, the results indicated that the unknown mutation in M577 generates a synthetic protein-*O-*mannosylation phenotype in combination with a *pmt1* but not a *pmt2* mutation.

To identify the mutation in M577, its *pmt1* derivative was transformed with a genomic bank in vector YEp13 and 80,000 transformants were screened for re-appearance of sensitivity to K1 killer toxin. Among 78 initial transformant isolates, 17 carried the complementing activity on a genomic insertion within the bank plasmid. Two genomic loci occurred repeatedly in overlapping inserts (Fig 1B): 5 plasmids contained a region close to the centromere of chromosome XIV and 4 plasmids contained a region close to the centromere of chromosome IX. In both cases, the overlapping clones had a single gene in common: the chromosome XIV clones contained *DOM34*, while the chromosome IX-clones contained *YIL001w*.

To clarify if the K1 killer-resistance phenotype in strain M577 *pmt1* was caused by mutation of *DOM34*, we introduced *pmt1*, *dom34* and *yil001w* mutations singly or in combination into the genetic background of the parental strain YE449. At variance with an initial report [16] but in agreement with a subsequent report [15] we found that *dom34* single mutants did not show significant growth defects. Importantly, we detected that the *pmt1 dom34* mutant but not the *pmt1 yil001w* mutant was killer-resistant, resembling the M577 *pmt1* strain (Fig 1Ab). Transformation with either *PMT1-*, *DOM34-* or *YIL001w-*overexpressing vectors restored killer-sensitivity of the *pmt1 dom34* mutant (Fig 1Ac). These results suggested that in the parental strain M577, the *DOM34* gene is mutated, while *YIL001w* may represent an extragenic suppressor of the *pmt1* and *dom34* mutations. In support of this conclusion we found that a diploid constructed from haploids M577 *pmt1* and W21 (YE449 *pmt1 dom34*) was unable to sporulate, as expected for a homozygous *dom34* diploid [15]. Furthermore, sequencing of the *DOM34* ORF in mutant M577 revealed that it is mutated by insertion of a single T residue following position 366 generating a UAA stop codon leading to a truncated protein of 122 residues.

### Dom34 and Pmt1 cooperate to increase hygromycin B resistance

The above results had suggested that in *S*. *cerevisiae*, the activities of specific elements involved in translation (Dom34) and protein-*O*-mannosylation (Pmt1) are functionally linked. We subsequently found that this cooperation not only prevents resistance (to killer toxin K1) but also enhances resistance to hygromycin B (HygB). HygB is an aminoglycoside antibiotic known to block translation [25], which is particularly active to block growth of glycosylation mutants [26]. *dom34 pmt1* double mutants were significantly more sensitive than the *pmt1* single mutant, although the *dom34* single mutant did not show any sensitivity phenotype (Fig 1C).

To generalize Dom34-Pmt1 functional interactions and because *S*. *cerevisiae* contains a *DOM34* homolog (YCL001W-B) with unknown activity, we also studied the single *DOM34* gene in the human fungal pathogen *C*. *albicans* that contains a family of well-studied Pmt proteins [3]. A homologue of *S*. *cerevisiae DOM34*, *ORF19*.*2419* in the *C*. *albicans* genome encodes a protein with 36%, 39% and 40% sequence identity to Dom34 proteins of *S*. *cerevisiae*, *S*. *pombe* and human, respectively. Sequence similarities are observed in the 3 domains of these proteins including a Sm-fold in domain 1 and a sequence in domain 3, which were both suggested to bind RNA (S1 Fig [13,15]). Compared to its homologues, the CaDom34 protein lacks a potential NLS sequence (position 173–177 in ScDom34p). Strains were constructed that lack both alleles of *CaDOM34* in the wild-type background (SK47), in the *pmt1* background (SK24) and in the *pmt5* background (JH8-5-11) (disruption scheme in S2 Fig). Phenotypes were determined using mutant strains, in which *URA3* was reconstituted at its authentic locus [27].

Similar to *S*. *cerevisiae*, a homozygous *dom34* single mutation did not generate significant growth or morphogenetic defects in *C*. *albicans*; furthermore, this mutant was found not to be supersensitive to numerous tested antibiotics or inhibitors including HygB. On the other hand, the HygB-supersensitive phenotype of the *pmt1* mutant was significantly increased by an additional *dom34* mutation (Fig 1C). Thus, evidence in both yeast species supported a functional link between Dom34 and Pmt1 proteins to generate HygB resistance. However, for *S*. *cerevisiae* it cannot be excluded that the *DOM34* paralog *YCL001W-B* contributes to this phenotype. Therefore, and because of its importance as a human pathogen, we focused subsequent analyses on the *C*. *albicans DOM34* gene.

### Overexpression of *DOM34* suppresses *pmt1* mutant phenotypes

**Fig 2:**
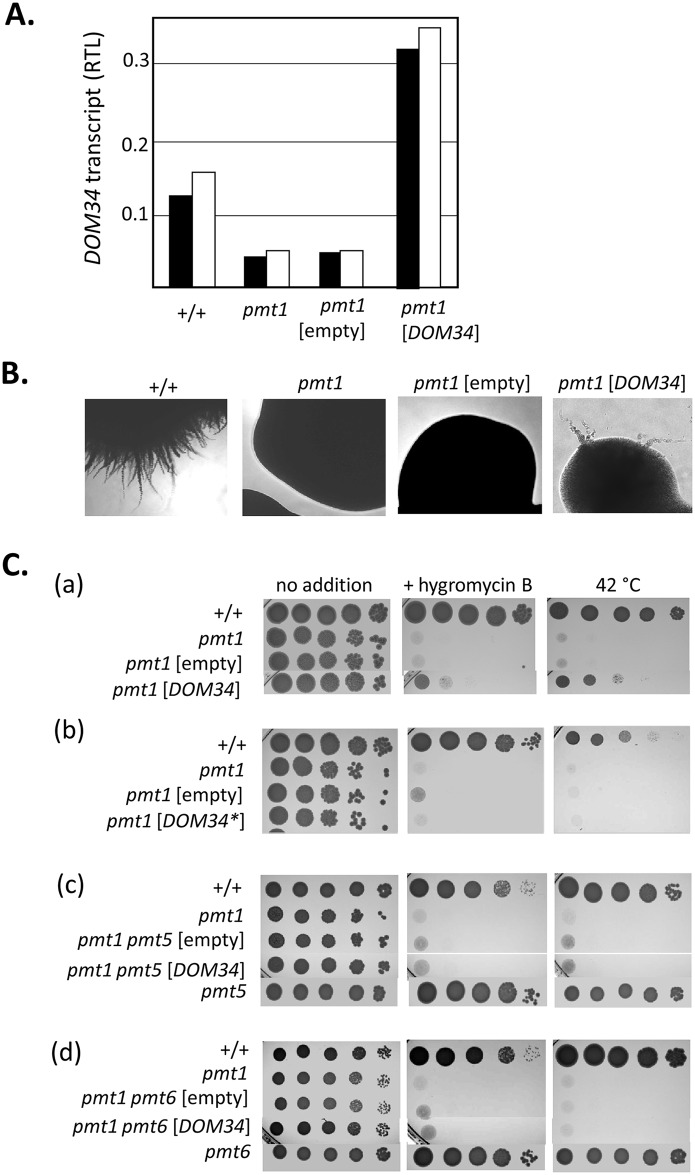
*DOM34* overexpression suppresses *pmt1* phenotypes. **(A)** Relative *DOM34* transcript level (RTL). Total RNA of strains CAF2-1 (+/+), SPCa2 (*pmt1*/*pmt1*)**,** CAP1-3121[pSP38] (*pmt1*/*pmt1*[empty vector] and CAP1-3121[pSK2] (*pmt1*/*pmt1*[*DOM34*]) was isolated and amounts of the *DOM34* transcript were determined by qPCR using *ACT1* as the reference transcript. Two independent biological replicates of each strain were assayed. **(B)** Hypha formation of representative colonies grown on Spider medium for 2–5 d at 37°C. Strain designations as in A. **(C)** Effects of *DOM34* overexpression on *pmt* mutant phenotypes. Serially diluted cultures of strains were spotted and grown on YPD agar at 30°C without or with 200 μg hygromycin B, or at 42°C without additions. *pmt1* single mutant host strains were transformed with expression vector pSK2 encoding wild-type Dom34 protein (a) or with pSK2mut encoding a E21A variant of Dom34 (Dom34*) (d). In addition, transformants of double mutant strains *pmt1 pmt5* (b) and *pmt1 pmt6* (c) were tested. Strains P15-274 (*pmt1*/*pmt1 pmt5*/*pmt5*), P15-274-1[pSP38] (*pmt1*/*pmt1 pmt5*/*pmt5*[empty vector], P15-274-1[pSK2] (*pmt1*/*pmt1 pmt5*/*pmt5*[*DOM34*]) were compared (b); in addition, strains CPP1121[pSP38] (*pmt1*/*pmt1 pmt6*/*pmt6*[empty vector]) and CPP1121[pSK2] (*pmt1*/*pmt1 pmt6*/*pmt6*[*DOM34*] were compared (c). Single mutant strains SPCa2 (*pmt1*/*pmt1*), SPCa10 (*pmt5*/*pmt5*), SPCa8 (*pmtD6*/*pmt6*) and the wild-type strain CAF2-1 (+/+) were used as reference strains.

The genetic interaction of *dom34* and *pmt1* mutations in *C*. *albicans* prompted experiments to study effects of *DOM34* overexpression in this fungus. The *DOM34* transcript level was determined by qPCR and showed an equal amount in homozygous *pmt4*, *pmt5*, *pmt6* and heterozygous *pmt2/PMT2* mutants as in the wild-type strain CAF2-1 but surprisingly, a 2–3 fold lower level in the *pmt1* mutant (Fig 2A; S3 Fig). A transformant of the *pmt1* mutant carrying plasmid pSK2 (*MET3p-DOM34*^*FLAG*^) that was grown in SD medium contained about sixfold higher *DOM34* transcript levels than the untransformed strain and about threefold higher levels than the wild-type strain. Thus, using pSK2, a moderate overexpression of *DOM34* was achieved in *C*. *albicans*.

Overexpression of *DOM34*^*FLAG*^ was able to rescue several known *pmt1* mutant phenotypes [3]. The inability of *pmt1* mutant colonies to form hyphae was partially suppressed by *DOM34* overexpression, which was observed in > 80% of the colonies (Fig 2B). Furthermore, the sensitivity of *pmt1* mutants to HygB and to high temperature (42°C) was also partially suppressed (Fig 2Ca). Interestingly, however, suppression was not achieved using a derivative of pSK2 carrying a point mutation in the *DOM34* ORF that encodes the E21A variant Dom34 protein (Fig 2Cb). It has been suggested that this residue is important for RNase activity of Dom34 proteins [18].

We asked next if the suppression of *pmt1* mutant phenotypes by *DOM34* overexpression depended on the Pmt isoforms remaining in this strain. For this purpose, we constructed *pmt1 pmt5* and *pmt1 pmt6* double mutants, which both showed the supersensitive *pmt1* mutant phenotype (Fig 2Cc and 2Cd), while single *pmt5* or *pmt6* mutants are not supersensitive to HygB [3]. Interestingly, double mutant transformants carrying the *DOM34* overexpression plasmid pSK2 did not show any recovery of *pmt* mutant phenotypes (Fig 2Cc and 2Cd). This result indicates that Pmt5 and Pmt6 isoforms are required for the observed rescue by Dom34 overproduction. The relevance of the Pmt2 and Pmt4 isoforms could not be tested in this manner, because *PMT2* is an essential gene and *pmt1 pmt4* double mutants are not viable [3]. These experiments also revealed that unexpectedly, a *C*. *albicans* strain lacking all members of the Pmt1 subfamily (Pmt1 and Pmt5) is fully viable, although in *S*. *cerevisiae* heteromeric Pmt1-Pmt2 or Pmt1-Pmt5 complexes have been described to be essential for growth [28].

### Dom34 stimulates Pmt protein but not transcript levels

**Fig 3:**
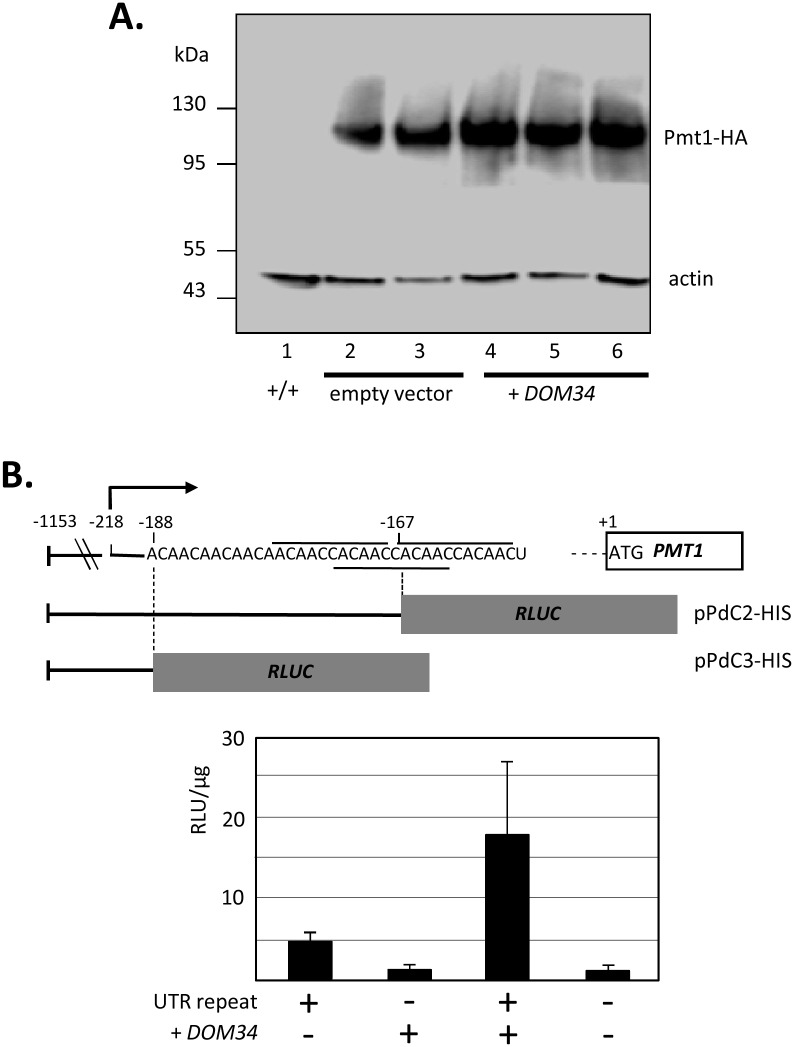
*Dom34* overexpression upregulates Pmt1 amounts and depends on 5′-UTR of *PMT1*. **(A)** Pmt1 amounts. Two independent strains of CIS23[pSP38] (*PMT1*/*PMT1*^HA^ [empty vector]) were compared with three independent strains of CIS23[pSK2] (*PMT1*/*PMT1*^HA^ [*DOM34*]); strain CAF2-1 (+/+) was used as negative reference strain. 5 μg of crude extract protein were separated by SDS-PAGE (10% acrylamide) and immunoblots were probed by rat anti-HA antibody (1:1000) and mouse anti-actin antibody (1:1000) followed by reaction with POD-coupled anti-rat and anti-mouse antibodies (1:20000). The signals of tagged Pmt1^HA^ and actin are indicated. **(B)** Importance of 5′-UTR on *PMT1* regulation. The 5′-start of the *PMT1* transcript at position -218 is indicated by the kinked arrow. Plasmids containing the *PMT1* promoter either including the 5′-end of the UTR from position -218 to -167 containing one of three 11-mer repeats (pPdC2-HIS) (under-/overlined sequences) or lacking this 5′-UTR sequence (pPdC3-HIS) in fusion to the *RLUC* reporter gene were integrated into the *PMT1* promoter of *C*. *albicans* strain RM1000 by transformation. These strains were additionally transformed with vectors pSK2 for *DOM34* overexpression or with the pSP38 empty control vector. Protein extracts of three independent double transformants were tested for luciferase activity, which was calculated as relative light units (RLU) per μg of protein.

Conceivably, Dom34 could have suppressed Pmt1 deficiencies by several mechanisms, especially by increasing transcription/transcript levels of several *PMT* genes including *PMT5* and/or *PMT6*, as suggested by the above experiments (Fig 2C). To explore this notion, we determined transcript levels in the *DOM34* overexpression strain and found that none of the *PMT* transcript levels was increased (S4A Fig). In addition, the absence of Dom34 did not decrease *PMT* transcript levels, while *PMT2/PMT4* transcript levels were increased in the *pmt1 dom34* double mutant, as described for the *pmt1* single mutant [6] (S4B Fig). Thus, increases and decreases of Dom34 levels were not related to *PMT* transcript levels. To explore effects on Pmt protein levels a *C*. *albicans* strain was constructed, in which one *PMT1* allele was fused to sequences encoding the hemagglutinin (HA)-epitope (Fig 3), which was subsequently transformed with the *DOM34* overexpression vector pSK2 or the corresponding empty vector. This strain produced considerably higher Pmt1^HA^ protein levels as compared to a transformant carrying an “empty” control vector (Fig 3A). Scanning of band intensities revealed that *DOM34* overexpression increased the mean Pmt1-HA/actin ratio 1.78 fold (*p = *0.025).

The 5′-UTR of the *PMT1* transcript encompasses 190 or 218 nt [29,30] and contains an intriguing CA/AC-rich sequence, which is ordered into three overlapping 11-mer ACAACCACAAC repeats between nt -157 to -179 (Fig 3B, top). To examine if this sequence is involved in overproduction of the Pmt1 protein by *DOM34* overexpression we generated genomic fusions containing two different lengths of the *PMT1* upstream region joined to the *RLUC* reporter gene. One fusion did not contain most 5′-UTR sequences (pPdC3-HIS), while the second contained one full and one half of the 11-mer repeat (pPdC2-HIS) (Fig 3B top). These strains were transformed with the *DOM34* overexpression plasmid pSK2 or a control plasmid and luciferase activity of the double transformants was determined. The results revealed that the construct containing 5′-UTR sequences including the 11-mer repeat was stimulated significantly by *DOM34* overexpression, while no activation occurred for the construct lacking UTR sequences (Fig 3B bottom).

As stated above, the *C*. *albicans* Dom34 protein lacks a consensus NLS sequence, suggesting that its primary action takes place outside of the nucleus. Furthermore, differential centrifugation of cell extracts identified HA-tagged Dom34^HA^ to a large extent in the soluble fraction (cytoplasm) (S5 Fig). Collectively, the results suggest that Dom34 overproduction stimulates translation of Pmt proteins. The site of the Dom34 stimulatory activity appears to lie in a specific sequence within the 5′-UTR of target transcripts, as exemplified by the Dom34-mediated regulation of the *PMT1-RLUC* fusion.

### Dom34 is required for efficient translation of the *PMT1* transcript

**Fig 4:**
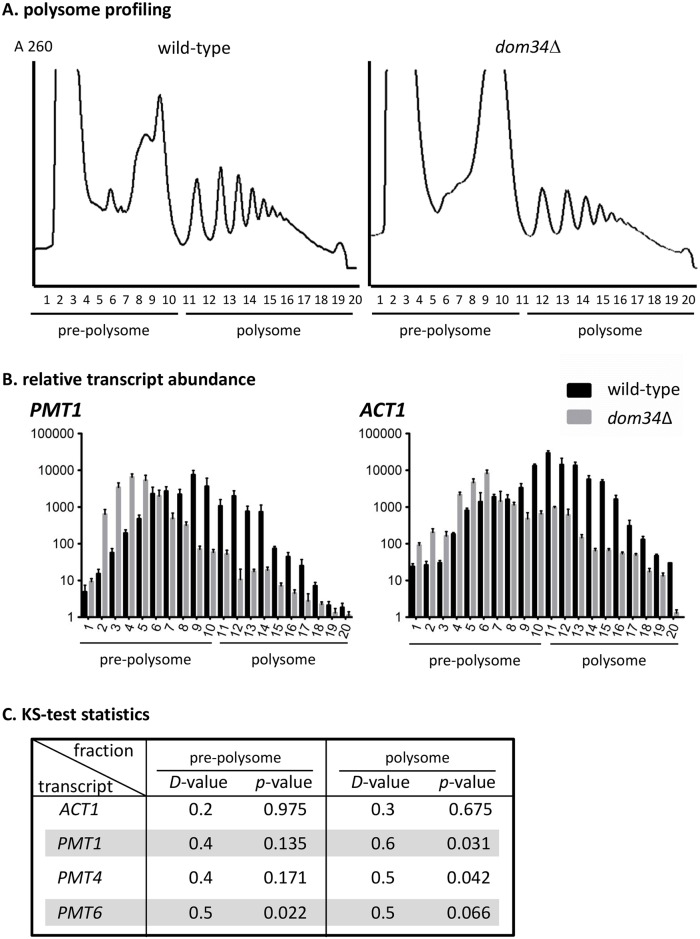
Transcript fractionation on polysome gradients. **(A)** Cellular extracts of strain CAF2-1 (wild-type) and *dom34* mutant JH47-2 were centrifuged in a 10–50% sucrose gradient, which was subsequently fractionated. Nucleic acids in gradient fractions were detected by absorbance (A_260_). Note that pre-polysome fractions contain 40S, 60S and 80S ribosomal RNA. **(B)** Occurrence of *ACT1* and *PMT1* transcripts in gradient fractions. Transcripts were detected by qPCR after adding a known amount of an *in vitro* generated transcript of CaCBGluc as calibrator. Each bar represents the normalized mean *ACT1* or *PMT1* transcript level of two independent experiments including the standard error of the mean. **(C)** The Kolmogorov-Smirnov test was used to determine the distance “D-value*”* between the two distribution functions of the wild-type strain CAF2-1 and *dom34* mutant for polysomal and pre-polysomal fractions of *PMT1*, *PMT4*, *PMT6* and *ACT1*, respectively. Statistical relevance is indicated by the calculated *p-*value.

We next carried out polysome analyses to establish the role of Dom34 in translation of *PMT1* and the *ACT1* housekeeping transcripts. For this purpose, cellular lysates of the control strain CAF2-1 and the *dom34* mutant JH47-2 were separated by sucrose gradient centrifugation to establish polysomal profiles (Fig 4A). Profile comparisons of both strains revealed that pre-polysomal fractions containing 40S, 60S and 80S rRNA were more pronounced in the *dom34* mutant than in the control strain; furthermore, polysomal peaks were lower in the mutant and decreased at >2n polysomes, whereas in the control strain, the 3n peak was even greater than the 2n peak. These results indicate that in the *dom34* mutant, translational efficiency is generally but moderately reduced.

To examine the efficiency of *PMT1* translation in control and *dom34* mutant cells, the fractions of the polysomal gradient were examined for the presence of the *PMT1* transcript by RT-qPCR, using a spiked-in control RNA as a reference. The result indicates clearly that the *PMT1* transcript in the *dom34* mutant is found predominantly in the pre-polysomal fraction, while in the control strain, significantly higher amounts reside in the polysomal fractions (Fig 4B). To establish if lower translational efficiency in the mutant concerns only the *PMT1* transcript, we also examined the transcript profile for the *PMT4* and *PMT6* transcripts, which also were enriched in the polysomal fraction in the presence of Dom34 (S6A Fig) indicating that Dom34 positively regulates the translation of several *PMT* transcripts. In comparison, translation of the transcript for the housekeeping gene *ACT1* appeared less affected by Dom34. These effects were quantitated the Kolmogorov-Smirnov test [31 (Fig 4C). By this algorithm, the calculated Dom34-dependent enrichment of transcripts in the polysomal fraction showed a statistically significant increase for the *PMT1* transcript (*D* = 0.6, *p*-value = 0.031), the *PMT4* transcript (*D = *0.5, *p = *0.042) and for the *PMT6* transcript (*D* = 0.5, *p* = 0.066); in contrast, a lower and insignificant enrichment was calculated for the *ACT1* transcript (D = 0.3, *p* = 0.675) (Fig 4C).

Collectively, the results suggest that Dom34 generally enhances but is not absolutely required for translation in *C*. *albicans*. The degree of Dom34-mediated translational enhancement differs between transcripts and may particularly affect specific groups of transcripts including transcripts for different Pmt isoforms. Interestingly, the 5′-UTR of all *PMT* transcripts (but not the 5′-UTR of the *ACT1* transcript) contains at least one CAAC motif, which in the above-described ACAACCACAAC repeat region of the *PMT1* 5′-UTR occurs eight times (S6B Fig). Conceivably, the positive action of Dom34 on translation of *PMT* transcripts is mediated by this sequence.

### Dom34 regulates *in vitro* translation via the *PMT1* 5′-UTR

**Fig 5:**
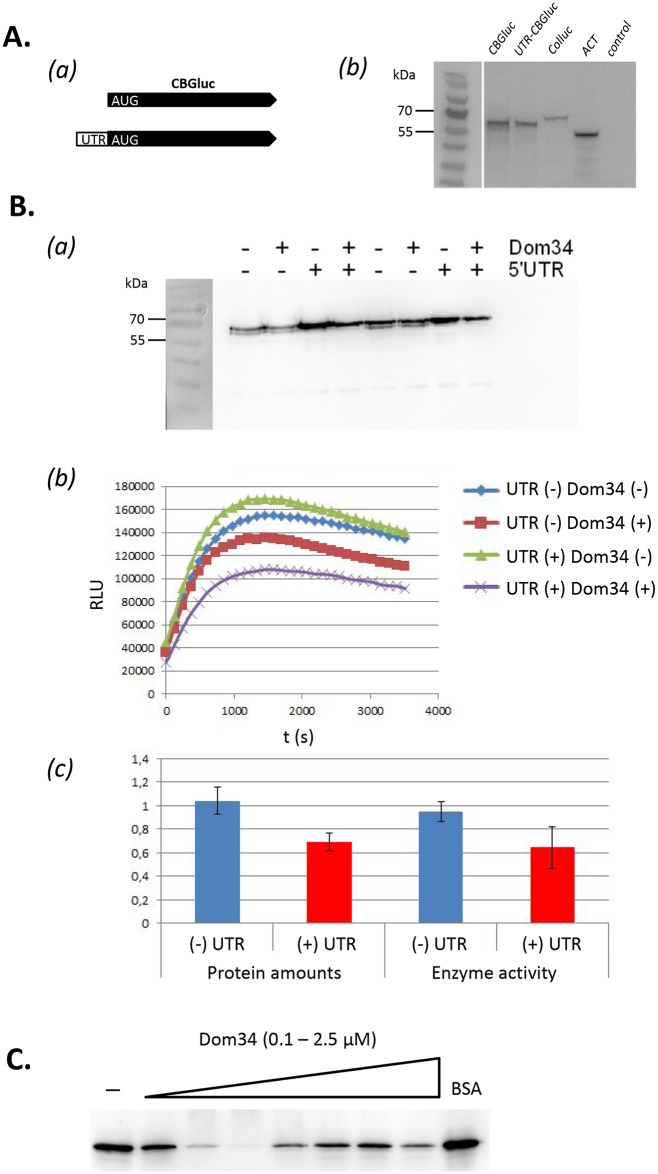
Dom34 inhibits translation when *PMT1* 5‘-UTR is present. **(A)** Identical sizes of CBGluc proteins produced in an *in vitro* rabbit reticulocyte translational system using RNA templates containing or not containing the 5‘-UTR of *PMT1*. *(a)* Scheme of RNA templates, *(b)* Protein products derived from *CBGluc* RNA without (*CBGluc*) or with (*UTR*-*CBGluc*) the 5′-UTR. Equal amounts of *in vitro* transcribed RNA were translated and labeled by incorporation of biotinylated lysine in the rabbit reticulocyte lysate; proteins were separated by SDS-PAGE and detected by using HRP-conjugated streptavidin. Further lanes contain protein products of control RNAs (Promega) showing 61 kDa *Coleoptera* luciferase (*Colluc*) and 42 kDa mouse ß-actin (Ambion) (*ACT*) and a control without RNA template (*control*). **(B)** Dom34 regulates *in vitro* translation. *(a)* CBGluc production as in (A) using no or 2.5 μM final concentration of Dom34, in the presence or absence of the 5′-UTR, as indicated. *(b)* Time course of luminescence emitted by the *in vitro* translated CBGluc, *(c)* comparisons of CBGluc protein amounts and CBGluc enzyme activities (luminescence peaks at 1332 sec) in the presence of Dom34. Three independent experiments were analyzed; the activity of samples without added Dom34 was set to 1. **(C)** Concentration dependence of *in vitro* translation of *CBGluc* transcript containing the 5‘-UTR by increasing amounts of Dom34 protein.

Recombinant Dom34 (S7 Fig) was added to a rabbit reticulocyte *in vitro* translation system, using RNA carrying the coding region for click beetle green luciferase (CBGluc), either containing or not containing the *PMT1* 5′-UTR (Fig 5Aa). As expected, protein products of identical molecular masses of 60 kDa were obtained for both proteins (Fig 5Ab). Additional experiments indicated that the presence of the 5′-UTR augmented CBGluc biosynthesis, while added Dom34 reduced production, if the 5′-UTR was present, but not in its absence (Fig 5Ba). These effects were quantitated by measuring CBGluc luminescence of the samples, which demonstrated that in the presence of Dom34, for RNA containing the 5′-UTR, enzyme activity was decreased by about 40% in three independent measurements (Fig 5Bb and 5Bc). Next, using translational assays containing varying amounts of Dom34 protein, it was shown that already at 0.1 μM, Dom34 reduces translation efficiency significantly, while it fully inhibits translation at 0.25 μM. At higher concentrations of Dom34, CBGluc production increases and again to diminishes at 2.5 μM. These results demonstrate clearly that Dom34 is able to strongly influence translational activity via the *PMT1* 5′-UTR sequence. Use of a heterologous *in vitro* system may explain the concentration-dependent, negative rather than the expected positive action of Dom34 on translation.

### Dom34 is able to bind and cleave endonucleolytically the *PMT1* 5’-UTR at distinct sites

**Fig 6:**
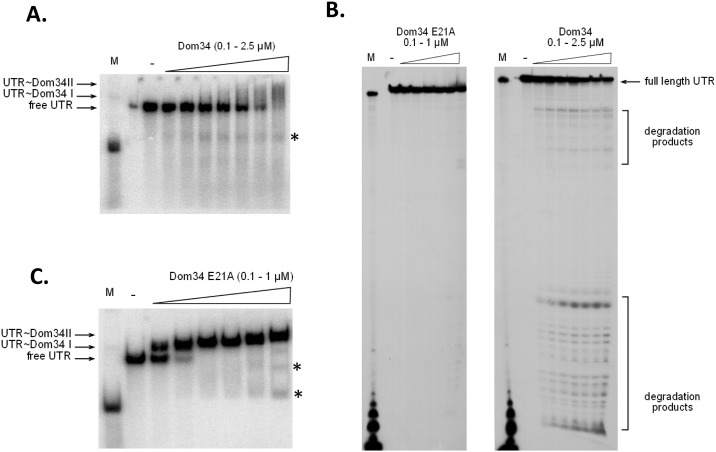
Dom34 is able to bind the *PMT1* 5‘-UTR and cleave at distinct sites. Radioactive 3′ end-labeled RNA was incubated with increasing amounts of recombinantly produced Dom34 or Dom34^E21A^ proteins and after complex formation samples were split and analysed either by 6% native PAGE **(A, C)** or by 10% denaturing PAGE **(B)**. Dom34 or Dom34 E21A were present in final concentrations of 0.1/0.15/0.25/0.3/0.6/1/2.5 μM, while BSA as a specificity control was added also at 2.5 μM final concentration. For comparison of running behaviour under native and denaturing conditions 6S RNA from *E*. *coli* was loaded on the gel (M). Two UTR-Dom34 complexes (I, II) were observed under native separation conditions **(A, C)**, which also revealed potential RNA degradation fragments (unbound or bound to Dom34) (asterisks). Under denaturing conditions Dom34 but not the Dom34 E21A variant generated specific UTR degradation fragments **(B)**.

To clarify the mechanisms how Dom34 influences translation we carried out electrophoretic mobility shift assays (EMSA) with recombinant Dom34 of the complete *PMT1* 5’-UTR sequence. The 5′-UTR was obtained by run off *in vitro* transcription using T7 polymerase using pRG01 as the template, which was cut by *Bgl*II at the 3′-end of the UTR. The 3′-[^32^PpCp] end-labelled RNA was incubated with increasing amounts of Dom34, separated by native PAGE (5% acrylamide) and examined by autoradiography. The 5′-UTR RNA migrated very slowly compared to the control 6S RNA from *E*. *coli* which is not much shorter and has a well-known compact secondary structure [32] (Fig 6A). This suggests a more bulky secondary structure for the *PMT1* 5’-UTR. At concentrations above 0.3 μM, the binding of Dom34 to the UTR became visible resulting in two retarded complexes (I and II). The second complex appeared at high Dom34 concentrations suggesting that multiple proteins are bound to one RNA molecule. At 2.5 μM of Dom34, no free RNA remained suggesting that all of the UTR was bound or degraded; in contrast, little binding of Dom34 (or Dom34^E21A^) to the 6S control RNA was observed (S8A and S8B Fig). Interestingly, starting already at very low concentrations of Dom34 (150 nM) some smaller-size degradation fragments of the UTR were observed (asterisks). Because the degradation products did not appear as sharp bands during native gel electrophoresis, we analyzed the same samples by denaturing gel electrophoresis (Fig 6B, right panel). Autoradiography clearly showed that already at 0.1 μM Dom34 the 5′-UTR is partially degraded and that the degradation products have distinct lengths of 1 to 40 nt and around 100 to 120 nt suggesting endonucleolytic cleavage rather than 3’ or 5’ exonucleolytic degradation. Exonuclease-mediated degradation from the 3′- end would have removed the 3′-labeling, which would have decreased amounts of full-length UTR RNA, while degradation from its 5′-end would have generated a smear of cleavage products. However, the results of denaturing gel electrophoresis indicate that the slight decrease of full-length UTR occurring at Dom34 concentrations > 0.6 μM is solely due to endonucleolytic fragmentation at specific sites. Under denaturing separation conditions protein-UTR interactions are disturbed, revealing the presence of full-length UTR that is not seen under native conditions. This result also provides an indirect proof for UTR binding by Dom34 at higher concentrations.

The 5′-UTR structure predicted by the RNAfold program (http://rna.tbi.univie.ac.at/cgi-bin/RNAfold.cgi) indicated that it contains several single-stranded regions including the presumed Dom34-binding CA/AC-rich sequence, as well as two double-stranded regions that compact the structure (S9A and S9B Fig). In the absence of Dom34, cleavage with single strand-specific RNase U2 confirmed that the CA/AC-region is unpaired, while in the presence of Dom34, this region was protected from RNase digestion, consistent with Dom34 binding in this region (S9C Fig). Furthermore, besides cutting the 5′-UTR at position 80–100 from 5′-end, binding of Dom34 made paired regions more accessible to RNase attack, thus indicating that significant rearrangement of the 5′-UTR had occurred during Dom34 binding.

Specific sequences in Dom34 homologs have been suggested to be important for the translational functions of Dom34 [17–20, 33]. A conserved glutamic acid residue in domain 1 (E21 in CaDom34) has been suggested to be important for RNase activity [18] and the importance of E21 for CaDom34 function was already shown above by the demonstration that the E21A variant is unable to suppress *pmt1* phenotypes (Fig 2C). To test effects of the E21A mutation on binding or cleavage of the *PMT1* UTR the Dom34-E21A variant was recombinantly produced in *E*. *coli*. Binding of Dom34-E21A to purified 5’-UTR was observed at very low protein concentrations (> 0.1 μM Dom34); estimation of an apparent K_D_ of about 100 nM deduced from these results demonstrates the very high affinity of Dom34-E21A to the UTR RNA, which is greater than the affinity observed for the wild-type Dom34 protein (Fig 6C). Importantly, minimal UTR cleavage is seen at concentrations > 0.6 μM of Dom34-E21A. The denaturing gel confirms this result, because only at 1 μM Dom34-E21A minimal degradation products become visible, while the amount of full-length RNA is not changed significantly (Fig 6B, left panel).

We conclude that Dom34 is an RNA binding protein, which favours certain RNA targets including the *PMT1* 5′-UTR. Furthermore, Dom34 has the capacity to endonucleolytically cleave bound target RNA and requires its E21 residue for this function.

### Dom34 binds to a 5′-UTR oligonucleotide

**Fig 7:**
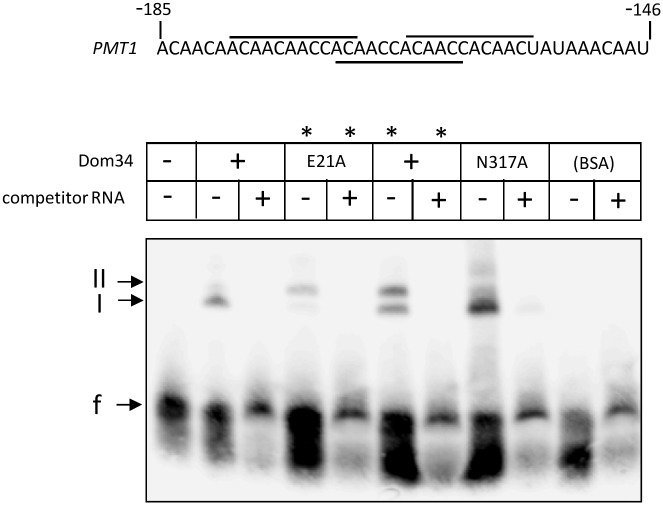
Dom34 binding to the 5′-UTR of the *PMT1* transcript. (Top) Sequence of oligonucleotide representing the 5′-end of the 5′-UTR of the *PMT1* transcript. (Bottom) EMSA of biotinylated 5′-UTR oligonucleotide in the absence or presence of *E*. *coli-*produced Dom34, its E21A variant and its N317A variant (protein/oligonucleotide molar ratio = 20). A specificity control reaction contains 2.5 μM BSA instead of Dom34. Unlabeled oligonucleotide in 100-fold excess was added as competitor in the indicated samples. The migration of unbound oligonucleotide (f) and two retarded complexes (I, II) was assayed by blotting of RNA separated by agarose gel electrophoresis onto a nylon membrane, which was developed by a chemiluminescent substrate to detected biotin. Binding reactions marked by an asterisk (*) were performed in the presence of 0.05% lauryl sarcosinate.

The above experiments had indicated that Dom34 stimulates translation (Fig 4) and that a specific sequence containing three overlapping 11-mer repeats within the 5′-UTR of the *PMT1* is involved in this function (Fig 3). To explore if Dom34 directly interacts with this sequence we tested interaction of a corresponding RNA oligomer containing the repeat sequence with Dom34 protein in EMSA. These experiments showed a single retarded complex (complex I) with wild-type Dom34, which in the presence of lauryl sarcosinate (asterisks) split into two retarded complexes (I, II) (Fig 7). In contrast, BSA as a control protein did not bind the RNA oligonucleotide and the labelled RNA oligomer could be competed out using a 100-fold excess of unlabeled oligomer. The E21A Dom34 variant showed even enhanced binding compared to native Dom34, supporting its binding behavior to the full-length 5′-UTR (Fig 6). Furthermore, the Dom34^N317A^ variant containing a mutation of a key residue in a sequence with high homology to RNA binding proteins [15] also bound to the 5′-UTR RNA oligomer as native Dom34. All Dom34 versions also led to a slight partial degradation of the labelled RNA oligonucleotide (smear emanating from free oligonucleotide); however, because a similar pattern was observed using the BSA control protein, it appears that no additional RNase activity is associated with Dom34.

## Discussion

By NGD, NRD and 80S release mechanisms, Dom34 and co-regulatory molecules including Hbs1 maintain sufficient numbers of ribosomes and thereby assure efficient translation in eukaryotes [10,14]. However, recent *in vivo* results have indicated that Dom34 affects ribosome occupancy at only 11% of all genes and it is yet unknown, why stalled ribosomes on some transcripts are resolved by Dom34, while other transcripts with similar structural impediments are not affected [13]. A restricted rather than a general function of Dom34 in translation was also suggested by the finding that *S*. *cerevisiae dom34* mutants do not show a general growth phenotype in all genetic backgrounds [15,16]. Thus, the target specificity of Dom34 for specific transcripts remains to be clarified. Results presented here indicate for the first time that Dom34 can serve as an RNA binding protein that could enhance translation of specific transcripts. These conclusions were obtained using the yeast *C*. *albicans* as experimental organism, which contains a single allele for a Dom34 protein with high similarity to orthologs in other organisms, while *S*. *cerevisiae*, because of its ancient genome duplication [34], contains in addition to *DOM34* a paralog (*YCL001W-B*) of unknown function.

Our results indicate on the one hand a general function of Dom34 on translation, since polysome gradients in the *dom34* mutant showed an increase of monosomes and decrease of polysomes, as compared to a wild-type strain. Furthermore, the abundance of the housekeeping *ACT1* transcript encoding actin was shifted slightly to the monosomal fraction in the *dom34* mutant suggesting reduced translation. However, the general translational effects of Dom34 appear to be moderate, since growth or morphogenesis of *C*. *albicans* was not affected in unstressed conditions. In contrast, in *pmt1* mutants with defective *O*-mannosylation that lack a major isoform of Pmt proteins [3], the contribution of Dom34 to growth phenotypes was clearly apparent. Protein-*O-*mannosylation is essential for fungal growth and its absence triggers the UPR response, because of the accumulation of underglycosylated, wrongly folded proteins in the ER lumen [6,7]. The *HAC1* transcript has recently been identified as a specific target of Dom34, which releases ribosomes stuck at the 3′-UTR [13]. One possible scenario explaining the genetic interaction of mutations in *DOM34* and *PMT1* is that Dom34, by its ribosome releasing function, assures efficient translation of the mature *HAC1* transcript. By this action, UPR responses could stimulate growth of *pmt1* mutant cells.

Aside from this general activity for maintenance of ribosome levels, our results suggest a specific stimulatory function of Dom34 on the translation of certain transcripts. As shown by the polysome profiling experiments, the translation of the *PMT1*, *PMT4* and *PMT6* transcripts were more strongly affected by the presence of Dom34 than that of the *ACT1* transcript. Furthermore, Dom34 binding to the 5′-UTR of the *PMT1* transcript and activation of a reporter gene by this sequence supported a direct positive role of Dom34 on the translational initiation of the *PMT1* transcript. This action is consistent with lowered Pmt1-mediated *O-*mannosylation of a heterologous protein (hIGF-1) during its massive overproduction in a *dom34* single mutant of *S*. *cerevisiae* [23]. In this overproduction condition, Dom34-mediated improvement of Pmt1 activity appears to be needed to obtain full *O-*mannosylation of target proteins. On the other hand, *DOM34* overexpression only rescued *pmt1* mutant phenotypes, if *PMT5* and *PMT6* genes were present, suggesting that the posttranscriptional stimulatory action of Dom34 is not exclusive for the *PMT1* transcript but applies to transcripts of several *PMT* genes, which was indeed confirmed for the *PMT4* and *PMT6* transcripts. Dom34 activity may be especially needed for translation of *PMT2* and *PMT4* transcripts, which in a compensatory response are upregulated in *pmt1* mutants [7]. On the other hand, stalled ribosomes were not found previously in a *dom34* mutant at any of the seven *S*. *cerevisiae PMT* transcripts [13], indicating that ribosome release by Dom34 is not a specific translational activation mechanism for *PMT* transcripts. The function of Dom34 as a relatively specific translational enhancer complements other posttranscriptional mechanisms in *C*. *albicans* that recently have been discovered to be essential for the biology and virulence of this fungus [35,36].

The ability of Dom34 to act as a RNA binding protein adds a new facet to the mode of action of Dom34 in eukaryotes. RNA binding was specific, because it did not occur with a control RNA, it was outcompeted efficiently and it occurred at low Dom34 concentrations. A stretch of residues in domain 3 of Dom34 had previously been shown to share high homology to RNA binding proteins [15,33]. However, mutation of a central residue in this sequence, N317 to N317A, did not affect binding of Dom34^N317A^ to the 5′-UTR oligonucleotide suggesting that this Dom34 sequence may be important for binding to the 3′-UTR but not to the 5′-UTR of transcripts. The predicted structure of the 5′-UTR, which was supported by limited RNase digestion, was found to contain several single-stranded regions, one of which comprised a CA/AC-rich region with three ACAACCACAAC repeats. Binding of Dom34 protected the 5′-UTR at this region from RNase digestion and Dom34 bound to a corresponding oligonucleotide, thus identifying the CA/AC-rich region as the Dom34 binding site. Interestingly, 5′-UTR regions of all five transcripts for *C*. *albicans* Pmt isoform (but not the 5′-UTR of the *ACT1* control transcript) contained at least one CAAC repeat (eight in the *PMT1* 5′-UTR), which may constitute the minimum requirement for Dom34 binding.

Dom34 did not show a major general RNase activity in our experiments, in agreement with Passos *et al*. [19], although distinct levels of specific degradation products of the bound 5′-UTR were detected. A Dom34 mutant, in which the conserved glutamic acid residue (E21 in CaDom34) important for *in vitro* RNase activity [18] was altered (E21A), strongly bound to the 5′-UTR but did not cause its endonucleolytic degradation, suggesting that E21 is relevant for its RNase activity. In agreement, rescue of *pmt1* phenotypes did not occur with overexpression of the *DOM34*^*E21A*^ allele, thus confirming the importance of the E21 residue for the function of Dom34. Dom34 binding to the CA/AC-rich sequence caused cleavage of the 5′-UTR by its RNase activity at a distant site, within predicted double-stranded regions causing a major structural alteration of the 5′-UTR. The mechanism, by which Dom34-mediated binding and cleavage of 5′-UTR sequences is able to stimulate translation of specific transcripts remains to be established. Possibly, the actions of Dom34 on the 5′-UTR structure could improve the accessibility of the AUG codon to ribosomal factors and subunits. Whatever the mechanism may be, the results suggest that in addition to its general action at the 3′-UTR to release stalled ribosomes, Dom34 may act at the 5′-UTR of specific transcripts including *PMT* transcripts to stimulate translational initiation in glycostress conditions.

## Methods and Materials

### *S*. *cerevisiae* strains

*S*. *cerevisiae* strains are listed in S1 Table. To disrupt *PMT* genes in strain YE449, a 3.3 kb *Xba*I-*Sac*I fragment of pDIS2, carrying *pmt1*Δ::*URA3*, or/and a 2.4 kb *Hin*dIII-*Bam*HI fragment of pBDis [24], carrying *pmt2*Δ::*LEU2*, were used for transformation, selecting prototrophs [37]. To disrupt *DOM34* in strain YE449, a fragment generated by PCR on plasmid pUG6 [38] was used for transformation, selecting G418-resistance (PCR primers ScDOM34 disrupt for /rev) Oligonucleotides are listed in S2 Table. Likewise, to disrupt *YIL001w*, a PCR fragment generated by primers ScYIL001w disrupt for/rev was used. Correct integration of the disruption cassettes was verified by Southern blottings.

### *C*. *albicans* strains

*C*. *albicans* strains are listed in S1 Table. For disruption of Ca*DOM34* its 5′- and 3′-regions flanking the ORF were inserted into pSFU1 to frame the *SAP2p-FLP* and *URA3* markers [39]. The *DOM34* 5′-region was amplified by genomic PCR on DNA of strain SC5314 using primers FPD34/RPD34 and the 3′-region was amplified using primers FDD34/RDD34. The resulting *DOM34* 5′-flanking region, as a *Sac*II-*Not*I fragment, and the 3′-flanking region, as a *Xho*I-*Apa*I fragment, were inserted into the respective sites of pSFU1 to generate pJB28. JB28 was cut with *Apa*I and *Sac*II and the large fragment was used for transformation of strain CAI4 or of *pmt1* mutant CAP1-3121 [40]. Correct integration in the resulting strains SK47 and SK24, respectively, was verified by diagnostic colony PCR and confirmed by Southern blottings, using the 5′-region flanking the *DOM34* ORF as the probe (S2 Fig). Removal of the *URA3* cassette and disruption of the second allele was carried out as described [41]. Following disruption of both *CaDOM34* alleles and eviction of the disruption cassette, *URA3* was reconstituted at its authentic locus by transformation with a genomic fragment [3] to generate strains JH47-1/2 and JH24-4/5. The *PMT5* gene was disrupted in the *dom34* background (strain SK47) or in the *pmt1* background (strain CAP1-3121) as previously described [3] to generate strains JH5-3-1 and P15-274, respectively.

To generate a *PMT1* gene encoding a C-terminally hemagglutinin (HA) epitope-marked Pmt1 protein PCR fragments were used containing the *sat1* selectable marker and flanked by regions of homology to *PMT1*. For tagging pSAT1-3HA was used as template, which was constructed by replacing the *URA3* gene in p3HA-URA [3] situated between *Pst*I and *Bgl*II sites with the *ACT1p-sat1-ACT1t*-cassette of pFC1 [42] on a *Pst*I to *Bam*HI fragment. PCR was done using primers CaPMT1del-for/-rev to generate a tagging fragment for Pmt1, which was chromosomally integrated by transformation of strain CAI4 selecting for nourseothricin resistance (*sat1*); resulting strain CIS23. For C-terminal HA-tagging of Dom34, an insertion fragment was generated by PCR using p3HA-URA as template and primers Dom34-HA-for/rev. The insertion fragment was transformed in strain CAI4 selecting for uridine prototrophy; resulting strains JHCa1-1 (-2). Correct integration of tagging cassettes was verified by diagnostic PCR of transformants.

### Growth conditions and screening method

*S*. *cerevisiae* and *C*. *albicans* strains were grown in complex YPD and synthetic SD media [37]. For hyphal induction of *C*. *albicans* the strains were grown for 3–4 days at 37°C on Spider-medium [43] or on 2% agar containing 5% horse serum.

To compare killer sensitivities of several *S*. *cerevisiae* strains, YPD agar containing methylene blue and buffered to pH 4.5 was autoclaved, cooled to 50°C and 17 μl of a saturated culture of the killer K1- secreting strain RC130 was added before pouring plates [44]. Strains to be tested were pre-grown on YPD medium and replica printed onto these plates and grown at 18°C for 4–7 days. Sensitive strains appeared blue at this time, while resistant strains remained white. To complement killer K1-resistance of strain M577 *pmt1* by genomic clones, we transformed it with a genomic *S*. *cerevisiae* bank in YEp13 [45] and obtained 80 000 transformants on SD minimal medium. Colonies of transformants were replica-printed onto SD/methylene blue/pH 4.5-medium containing killer K1 strain RC180. Blue colonies were picked, their plasmid was isolated and retransformed into M577 *pmt1*. Among 78 initial transformants, 17 transformants were identified, whose plasmids restored Killer-sensitivity upon retransformation. Insert ends in these plasmids were sequenced using primers YEp13-Bamflank-A/-B, flanking the *Bam*HI insertion site of YEp13.

### Yeast expression plasmids

The 7 kb *Bam*HI-*Xho*I fragment of pDM3 carrying a genomic *ScPMT1* fragment [46] was subcloned into YCplac111, to generate pSW20. Derivatives of YEp13 containing a 5.59 kb genomic insert carrying *ScDOM34* (pSW577/20) or containing a 4.55 kb genomic insert carrying *YIL001w* were used in some complementation experiments. Expression vectors encoding HA-tagged proteins were constructed by PCR amplification of ORFs and introducing them into YCpIF17 [47]. *DOM34* was amplified using primers ScDom34N/C, the 1.2 kb product was digested with *Eco*RI and *Pst*I and introduced into YCpIF17, resulting in plasmid pSW22. The *GAL1p-HA-DOM34* fragment of pSW22 was excised with *Xho*I and *Xba*I and inserted into YCplac111 (*Sal*I, *Xba*I) to generate pSW25. Likewise, *YIL001w* was amplified using primers YIL001wN/C, inserted into YCpIF17 to generate pSW21 and transferred into YCplac111 to generate pSW24.

To construct a *CaDOM34* expression vector its coding region was PCR amplified on gDNA using primers p1-DOM-FLAG and p2-DOM-FLAG. We then inserted the resulting *Pst*I-*Sph*I fragment downstream of the *MET3* promoter into a derivative of pFLAG-Met3 [48], which had been modified by adding the *CaARS2* replicator on an *Aat*II fragment [49]. The resulting plasmid pSK2 was modified further by oligonucleotide-directed mutagenesis to encode the Dom34-E21A variant; for this purpose, pSK2 was used as template with primers Dom34Mut21for/rev in the QuikChange protocol (Stratagene) to generate pSK2mut. Using anti-FLAG antibody the FLAG-tagged Dom34 protein could be identified as a 45 kDa protein in immunoblottings of cellular extracts, although several cross-reacting proteins prevented immunocytological analyses.

### Production of Dom34 in *E. coli*

The *C*. *albicans DOM34* ORF was inserted downstream of the T7 promoter, between the *Nde*I and *Xho*I restriction sites of expression vector pET22b (Invitrogen). The resulting vector pET22b-Dom34 encoded a Dom34 protein containing six histidine residues at its C-terminal end. The single CTG sequence encoding non-standard S288 in *C*. *albicans* was then altered to standard serine-encoding TCG by oligonucleotide-directed mutagenesis, using primers Dom34-Leu(mut)for/rev according to the QuikChange protocol (Agilent). The resulting vector pET22b-Dom34+ was modified further similarly to encode variants potentially important for the function of Dom34. For the E21A variant and the N317A variant, oligonucleotides Dom34Mut21fw/rev and Dom34Mut317fw/rev were used for mutagenesis. All expression vectors were verified by sequencing and transformed in *E*. *coli* strain Rosetta (Novagen).

Transformant cultures were grown to OD_600_ = 0.6 in LB medium containing 100 μg/ml ampicillin and 50 μg/ml chloramphenicol, 100 mM IPTG was added to induce the T7 promoter and cultures were incubated further at room temperature. Cell pellets were resuspended in buffer (50 mM TrisHCl/pH 7.9; 500 mM NaCl; 10% glycerol) containing protease inhibitors and disrupted by ultrasonication, followed by centrifugation (10,000 *x g* for 20 min). To assure the solubility of recombinant proteins, 0.5% lauryl sarcosinate was added to the buffer in some experiments. Soluble Dom34-His_6_ proteins in the supernatant were purified by affinity chromatography using Histrap Crude-Agarose columns (GE) using 5 mM, 50 mM and 250 mM imidazole for elution. Elution fractions were collected using a ÄKTAprime collector (GE Healthcare) and analyzed by SDS-PAGE (4–20% acrylamide) followed by Coomassie Blue staining or by immunoblotting using an HRP-coupled anti-His tag antibody (Qiagen) (S7 Fig). About 2 mg of Dom34 variants with at least 95% purity were obtained.

### qPCR

Relative transcript levels (RTL) of specific *C*. *albicans* genes were determined by quantitative reverse transcription PCR (qPCR) using the *ACT1* transcript as reference, as described [7,8]. Oligonucleotides designated RT (S2 Table) were used for this purpose.

### *In vitro* translation

For the *in vitro* generation of transcripts, plasmid pUC18-T7CBG was constructed, which contains the promoter of bacteriophage T7 upstream of the coding region for click beetle green luciferase (CBGluc). For this purpose, primers CBG-Stu/Bam for/rev were used to amplify the coding region for CBGluc from plasmid CBGluc-pMK-RQ [50], which was inserted into pUC18T7 [51]. The resulting plasmid was modified further by insertion of the *PMT1* 5′-UTR sequence (-218 to -1 [29]) that was amplified using primers PMT1_5′UTR/-rev BglII_long from gDNA (strain CAF2-1); the resulting plasmid was named pRG01. Plasmids were linearized by *Bam*HI (pUC18-T7-CBG) or *Bgl*II (pRG01) downstream of the CBGluc coding region and were used as template for *in vitro* run-off transcription using T7 RNA polymerase (Ambion SP6 *in vitro* transcription kit) according to Gildehaus *et al*. [51]). The resulting transcripts were used for *in vitro* translation in a rabbit reticulocyte lysate kit (Promega L4960) that incorporates biotinylated lysine in the protein products (Promega Transcend Detection System). Divergent from the manufacturer's protocol the reaction was reduced to a total volume of 20 μl with 0.75 μl Transcend^™^ Biotin-Lysyl-tRNA. For each reaction 4.9 fmol RNA (244 nM) and variable amounts of Dom34 were preincubated for 10 min at RT and the reaction was started by adding a premix of reticulocyte-lysate, amino acids and RNasin. After 100 min at 30°C, 1 μl was separated by SDS-PAGE (10% acrylamide), followed by reaction with horseradish peroxidase-coupled streptavidin, as described by the manufacturer. Furthermore, CBGluc luminescence was measured by adding 45 μl of water to 5 μl of the translation mix, followed by addition of 50 μl Chroma-Glo substrate (Promega). Measurements were done in wells of 96-microtiter plates using TriStar LB 941 luminometer (Berthold Technologies) for 1 sec, as described [50].

### Electrophoretic Mobility Shift Assay (EMSA)

Labelled RNA encompassing the 5′-UTR was obtained by multiple rounds of run-off transcription of pRG01 linearized by *Bgl*II and T7 RNA polymerase [48]. pUC18-T7-6S linearized by *Stu*I served to generate 6S control RNA [51]. After purification on agarose-gels followed by glass wool-elution, the radioactive labeling at the 3′-end was performed by T4-RNA ligase-catalyzed addition of ^32^P-pCp, as described [52]. If necessary, the labeled RNA was purified in a second step by electrophoresis on a 5% denaturing polyacrylamide gel. RNA-Dom34 complex formation was assayed by incubating 50–100 cps ^32^P-labeled RNA together with variable amounts of Dom34 for 10 min at 30°C, in 10 mM Tris-HCl/pH 8.0, 100 mM NaCl. Complexes were then challenged by adding of heparin (final concentration 50 ng/μl) for 10 min and separated on native 5% polyacrylamide gels or denaturing 10% polyacrylamide gels.

For EMSA of the *PMT1-*5′UTR RNA oligonucleotide, it was biotinylated using the Pierce RNA 3′ End Biotinylation Kit (Thermo Scientific) according to the instructions of the manufacturer. Protein-RNA binding assays were done using the LightShift Chemoluminescent RNA EMSA Optimization and Control Kit (Thermo Scientific). In a total volume of 20 μl, binding assays contained 20 nM biotinylated RNA oligonucleotide and 400 nM purified Dom34-His_6_ (or its E21A or N317A variants), which were supplemented in part by 2 μM unlabelled oligonucleotide. Following incubation at room temperature for 20 min, 5 μl of 5x REMSA loading buffer was added and the assay components were separated by native PAGE (6% acrylamide) and electroblotted onto a nylon membrane; the biotinylated RNA was fixed on the membrane by a short UV treatment and was detected using the Chemiluminescent Nucleic Acid Detection Module Kit (Thermo Scientific).

### Polysomal profiling

Cells of wild-type strain CAF2-1 and of *dom34*Δ mutant JH47-2 cells were grown exponentially in YPD media to OD_600_ 0.4–0.6. Preparation of cells and polysome gradients were performed as described by Garre *et al*. [53] with some modifications. A portion of the culture (80 ml) was recovered and chilled for 5 min on ice in the presence of 0.1 mg/ml cycloheximide (CHX). Cells were harvested by centrifugation at 6000 *x g* for 4 min at 4°C and resuspended in lysis buffer (20 mM Tris-HCl, pH 8, 140 mM KCl, 5 mM MgCl_2_, 0.5 mM dithiothreitol, 1% Triton X-100, 0.1 mg/ml CHX, and 0.5 mg/ml heparin). After washing, cells were resuspended in 700 μl of lysis buffer, a 0.3 ml volume of glass beads was added, and cells were disrupted by shaking on a Vortex Genie 2 (setting 8) using 6 cycles for 40 s at 6.5 ms^-1^. Between cycles cells were placed on ice for 5 min. Lysates were cleared by centrifuging twice for 5 min, first at 5,000 rpm, and then the supernatant was recovered and was centrifuged at 8,000 rpm. Finally, glycerol was added to the supernatant at a final concentration of 5%, before storing extracts at -70°C. Samples of 10–20 A_260_ units were loaded onto 10–50% sucrose gradients and were separated by ultracentrifugation for 2 h and 40 min at 35,000 rpm in a Beckman SW41 rotor at 4°C. Then, gradients were fractionated using isotonic pumping of 60% sucrose from the bottom, followed by a recording of the polysomal profiles by online UV detection at 260 nm (Density Gradient Fractionation System, Teledyne Isco, Lincoln, NE).

To analyze the RNA of the polysomal fractions, RNA from 200 μl of each fraction was extracted using GeneJet RNA extraction kit (STREK, Biotools). To each sample 1 μg of *in vitro* transcribed RNA (HiScribe^™^ T7 High Yield RNA Synthesis Kit, NEB) was added and used as spiked-in mRNA for normalization of the transcripts. After reverse transcription of the purified RNA (Maxima First Strand cDNA synthesis kit, Thermo Scientific) quantitative PCR (RT-qPCR) was performed using gene specific primer pairs to quantify mRNAs of *PMT1*, *PMT4*, *PMT6* and *ACT1*. For each fraction three technical replicates were assayed on a Mx3000P LightCycler (Stratagene), with 10 μl of cDNA, 4 μl EvaGreen QPCR-mix II (Bio-Budget) and 3 μl each of forward and reverse oligonucleotide primers (400 pmol/μl) in each reaction. The polymerase was activated at 95°C for 10 min, annealing was performed at 60°C for 15 s, extension at 72°C for 30 s and the denaturation step was performed at 95°C for 30 s in a total of 50 cycles.

For the statistical assessment of the difference between the transcript distributions in the reference strain CAF2-1 and the *dom34* mutant strain, the Kolmogorov-Smirnov test (KS-test) was performed [31]. The KS-test was computed using the *ks*.*test* CRAN package in the *R* statistical software environment.

## Supporting Information

S1 Fig Structure of Dom34 proteins. Dom34/Pelota sequences of *S*. *cerevisiae* (ScDom34), *C*. *albicans* (CaDom34), *D*. *melanogaster* (DmPelota) and *S*. *pombe* (SpDom34) are aligned. The positions of domains 1–3 is indicated by the arrows and the circled numbers. The region suggested as a RNA binding sequence in domain 3 is underlined. The conserved glutamate in domain 1, presumed to act in RNase activity, is indicated by the thick arrow.
(PDF)

S2 Fig Disruption of *DOM34* locus. **(A)** Scheme of wild-type *DOM34* locus, after disruption by the *FRT-URA3—FLP-FRT* cassette and after removal of the *URA3-FLP* sequences (top-to-bottom). **(B)** Southern blotting of genomic DNA in transformants. Total DNA was digested with *Cla*I and *Sal*I and blots were probed using a *DOM34* segment indicated by asterisks in (A). The fragment for the wild-type alleles is visible in lane 1, while the subsequent lanes demonstrate the course of disruption in derivative strains and the complete disruption of the two *DOM34* alleles in strain SK47 (lane 5).
(PDF)

S3 Fig *DOM34* transcript levels in *pmt* mutant strains. Total RNA of strains CAF2-1 (+/+), SPCa2 (*pmt1*/*pmt1*), SPCa4 (*pmt2*/*PMT2*), SPCa6 (*pmt4*/*pmt4*), SPCa10 (*pmt5*/*pmt5*) and SPCa8 (*pmt6*/*pmt6*) was isolated and the relative *DOM34* transcript level (RTL) was determined by qPCR using the *ACT1* transcript as the reference. Values obtained for two biological replicates are shown as black and white bars.
(PDF)

S4 Fig Influence of *DOM34* expression on *PMT* transcript levels. **(A)** Influence of *DOM34* overexpression was tested using strains CAF2-1 (+/+), SPCa2 (*pmt1*/*pmt1*), CAP1-3121[pSP38] (*pmt1*/*pmt1*[empty vector] and strain CAP1-3121[pSK2] (*pmt1*/*pmt1*[*DOM34*]). **(B)** Influence of *dom34* mutation was tested using strains CAF2-1 (+/+), SPCa2 (*pmt1*/*pmt1*), JH24-4 (*pmt1*/*pmt1 dom34*/*dom34*) and JH47-1 (*dom34*/*dom34*). Total RNA of all strains was isolated and relative transcript levels of the indicated *PMT* genes were determined by qPCR using the *ACT1* transcript as the reference. Values obtained for two independent biological replicates are shown as black and white bars.
(PDF)

S5 Fig Immunoblot of HA-tagged Dom34. Cells of strains JHCa1-1 (*DOM34/DOM34*^*HA*^) were fractionated by differential centrifugation. The periplasmic space (PE), crude extract (CE), pellet after centrifugation at 10,000 *x g* (P10; ER fraction), pellet after centrifugation of P10 supernatant at 100,000 *x g* (P100; Golgi fraction) and the corresponding supernatant (S100; cytoplasmic fraction) were obtained. Aliquots of each fraction were examined by SDS-PAGE (10% acrylamide) followed by immunoblotting using rat anti-HA antibody. The arrow indicates the migration of HA-tagged Dom34. As a control, an immunoblot of a crude extract of strain CIS23 (*PMT1/PMT1*^*HA*^), producing HA-tagged Pmt1, is shown.
(PDF)

S6 Fig Occurrence of *PMT4* and *PMT6* transcripts in polysome-gradient fractions. **(A)** The indicated transcripts were detected by qPCR after adding a known amount of an *in vitro* generated transcript of CaCBGluc as calibrator. Each bar represents the normalized mean *PMT4* or *PMT6* transcript level of two independent experiments including the standard error of the mean. **(B)** Incidence of “CAAC” and “ACCA” motifs in 5’-UTRs of *ACT1* and *PMT*-genes. The 5’-UTR sequences of *ACT1* (reference gene) and all *C*. *albicans PMT*-genes were analyzed for occurrence of the identified “CAAC” and “ACCA” motif (Fig 3B). Information about transcript start sites were taken from Tuch *et al*. (2010) and Bruno *et al*. (2010) [29,30]; the transcript with the longer 5’-UTR was chosen and respective sequences were obtained from CGD (http://www.candidagenome.org) assembly 21. Occurrence of the “CAAC” (underlined) and “ACCA” (line on top) motifs are highlighted in red at the indicated positions. The “CAAC” motif was identified in 5’-UTRs of all *PMT*-genes, but not in the 5’-UTR sequence of the reference gene *ACT1*.
(PDF)

S7 Fig Purification of Dom34-His_6_. *E*. *coli* Rosetta transformants carrying pET22b-Dom34+ were induced by IPTG and cell pellets were disrupted by ultrasonication. Soluble proteins in the cell extract were separated by affinity chromatography on Ni-NTA agarose, which was washed in buffer using 5 mM imidazole and eluted using 50 and 250 mM imidazole. Aliquots of fractions were separated by SDS-PAGE (4–20% acrylamide) and gels were stained using Coomassie Brilliant Blue R-250. Lanes show protein standards (S), cell pellet (Pe), flow-through (F), wash fractions (W1,2) and elution fractions (E1-5). The arrow marks the position of Dom34-His_6_.
(PDF)

S8 Fig Weak binding of native Dom34 (A) or the Dom34 E21A variant (B) to 6S RNA from *E*. *coli*. Radioactive 3‘ end-labeled 6S RNA was incubated with increasing amounts of Dom34; after complex formation samples were analysed by 6% native PAGE. As indicated Dom34 was present in final concentrations of 0.1/0.15/0.25/0.3/0.6/1 and 2.5 μM.
(PDF)

S9 Fig Sequence and structure of the *PMT1* 5′-UTR. **(A)** UTR RNA sequence generated by *in vitro* transcription of plasmid pRG01 (linearized by *Bgl*II) using T7 RNA polymerase. Two G residues are added to 218 nt UTR sequence at its 5′-end by T7 RNA polymerase, while AGATC at the 3′-end reflects the *Bgl*II sequence. The transcript start site at -218 was determined by Tuch *et al*. [29], while an additional start site at -190 was reported by Bruno *et al*. [30] (underlined U in sequence). The CA/AC-rich region (Dom34 binding site) is marked in red font. **(B)** Predicted folding structure of *PMT1* UTR. The RNAfold program (http://rna.tbi.univie.ac.at/cgi-bin/RNAfold.cgi) was used for prediction and results were depicted as a centroid structure drawing encoding base-pair probabilities (colour code showing probabilities of base-pairing or single strandedness in predicted paired and unpaired regions, respectively). Numbered black arrows indicated predicted events upon Dom34 binding: (1) Binding of Dom34 to single-stranded region containing CA/AC-repeats, (2) opening of paired region by binding of Dom34, (3) opening of paired region and cleavage by Dom34, (4) cleavage by Dom34. **(C)** RNAse cleavage experiments supporting the predicted actions of Dom34. 3′-[^32^P] end-labelled UTR RNA (50 cps) was incubated with RNases in the absence or presence of Dom34 for 1 min at 37°C; Dom34 was preincubated 10 min with the UTR before RNAse addition. Products were separated by 12% denaturing PAGE. Nucleotide positions are numbered from the UTR 5′-end as in B. UTR, no RNAse (lane 1); UTR with 1 U RNAse T1 cleaving at G residues indicated in the left margin (lane 2); UTR with 0.5 U RNAse U2 specific for RNA single strands (lane 3); UTR with 1 μM Dom34 (lane 4); UTR sequence ladder generated by partial hydrolysis with NaOH (lane 5); UTR with 0.25/0.5/1 U RNAse U2 (lanes 6–8); UTR incubated with 0.5 U RNAse U2 and 0.5/1/2.5 μM Dom34 (lanes 9–11). Note a prominent Dom34-mediated cleavage of the UTR around position 100. RNase U2 cleavage in position 30–60 (CA/AC-repeat region) confirms the predicted single-strandedness of the UTR in this position (lanes 7–9); Dom34 protects this region (lanes 10, 11), confirming its predicted binding at this site. Region 180–190 appears permanently single-stranded, while the predicted double-stranded regions 65–77 and around 170 become single-stranded upon Dom34 binding (lanes 9–11).
(PDF)

S1 Table Strains. (PDF)

S2 Table Oligonucleotides. (PDF)

## References

[1] Lengeler KB, Tielker D, Ernst JF. Protein-*O-*mannosyltransferases in virulence and development. Cell Mol Life Sci. 2008;65: 528–544. 10.1007/s00018-007-7409-z 17975704 PMC11131616

[2] Willer T, Valero MC, Tanner W, Cruces J, Strahl S. *O*-mannosyl glycans: from yeast to novel associations with human disease. Curr Opin Struct Biol. 2003;13: 621–630. 10.1016/j.sbi.2003.09.003 14568618

[3] Prill SK-H, Klinkert B, Timpel C, Gale CA, Schröppel K, Ernst JF. *PMT* family of *Candida albicans*: five protein mannosyltransferase isoforms affect growth, morphogenesis and antifungal resistance. Mol Microbiol. 2005;55: 546–560. 10.1111/j.1365-2958.2004.04401.x 15659169

[4] Gentzsch M, Tanner W. Protein-*O*-glycosylation in yeast: protein-specific mannosyltransferases. EMBO J. 1996;15: 5752–5759. 8918452 PMC452322

[5] Rouabhia M, Schaller M, Corbucci C, Vecchiarelli A, Prill SK-H, Giasson L, et al Virulence of the fungal pathogen *Candida albicans* requires the five isoforms of protein mannosyltransferases. Infect Immun. 2005;73: 4571–4580. 10.1128/IAI.73.8.4571-4580.2005 16040968 PMC1201229

[6] Cantero PD, Lengsfeld C, Prill SK-H, Subanovíc M, Román E, Pla J, et al Transcriptional and physiological adaptation to defective protein-O-mannosylation in *Candida albicans*. Mol Microbiol. 2007:64**:** 1115–1128. 10.1111/j.1365-2958.2007.05723.x 17501932

[7] Cantero PD, Ernst JF. Damage to the glycoshield activates *PMT-*directed *O*-mannosylation via the Msb2-Cek1 pathway in *Candida albicans*. Mol Microbiol. 2011;80: 715–725**.** 10.1111/j.1365-2958.2011.07604.x 21375589

[8] van Wijlick L, Swidergall M, Brandt P, Ernst JF. *Candida albicans* responds to glycostructure damage by Ace2-mediated feedback regulation of Cek1 signaling. Mol Microbiol., in press. 10.1111/mmi.13494 27589033

[9] Payne T, Hanfrey C, Bishop AL, Michael AJ, Avery SV, Archer DB. Transcript-specific translational regulation in the unfolded protein response of *Saccharomyces cerevisiae*. FEBS lett. 2008;582: 503–509. 10.1016/j.febslet.2008.01.009 18206654

[10] Doma MK, Parker R. Endonucleolytic cleavage of eukaryotic mRNAs with stalls in translation elongation. Nature. 2006;440: 561–564. 10.1038/nature04530 16554824 PMC1839849

[11] Shoemaker CJ, Eyler DE, Green R. Dom34:Hbs1 promotes subunit dissociation and peptidyl-tRNA drop-off to initiate no-go decay. Science. 2010;330: 369–372. 10.1126/science.1192430 20947765 PMC4022135

[12] Tsuboi T, Kuroha K, Kudo K, Makino S, Inoue E, Kashima I, et al Dom34:hbs1 plays a general role in quality-control systems by dissociation of a stalled ribosome at the 3' end of aberrant mRNA. Mol Cell. 2012;46: 518–259. 10.1016/j.molcel.2012.03.013 22503425

[13] Guydosh NR, Green R. Dom34 rescues ribosomes in 3' untranslated regions. Cell. 2014;156: 950–962. 10.1016/j.cell.2014.02.006 24581494 PMC4022138

[14] Bhattacharya A, McIntosh KB, Willis IM, Warner JR. Why Dom34 stimulates growth of cells with defects of 40S ribosomal subunit biosynthesis. Mol Cell Biol. 2010;30**:** 5562–5571. 10.1128/MCB.00618-10 20876302 PMC2976434

[15] Carr-Schmid A, Pfund C, Craig EA, Goss Kinzy T. Novel G-protein complex whose requirement is linked to the translational status of the cell. Mol Cell Biol. 2002;22: 2564–2574. 10.1128/MCB.22.8.2564-2574.2002 11909951 PMC133728

[16] Davis L, Engebrecht J. Yeast *dom34* mutants are defective in multiple developmental pathways and exhibit decreased levels of polyribosomes. Genetics. 1998;149: 45–56. 9584085 10.1093/genetics/149.1.45 PMC1460139

[17] Graille M, Chaillet M, van Tilbeurgh H. Structure of yeast Dom34. A protein related to translation termination factor Erf1 and involved in no-decay. J Biol Chem. 2008;283: 7145–7154. 10.1074/jbc.M708224200 18180287

[18] Lee HH, Kim YS, Kim KH, Heo I, Kim SK, Kim O, et al Structural and functional insights into Dom34, a key component of no-go mRNA decay. Mol Cell. 2007;27: 938–950. 10.1016/j.molcel.2007.07.019 17889667

[19] Passos DO, Doma MK, Shoemaker CJ, Muhlrad D, Green R, Weissman J, et al Analysis of Dom34 and its function in no-go decay. Mol Cell. 2009;20: 3025–3032. 10.1091/mbc.E09-01-0028 19420139 PMC2704154

[20] van den Elzen A.M., Schuller A., Green R., Séraphin B. Dom34-Hbs1 mediated dissociation of inactive 80S ribosomes promotes restart of translation after stress. EMBO J. 2014;33, 265–276. 10.1002/embj.201386123 24424461 PMC3989619

[21] Cole SE, LaRiviere FJ, Merrikh CN, Moore MJ. A convergence of rRNA and mRNA quality control pathways revealed by mechanistic analysis of nonfunctional rRNA decay. Mol Cell. 2009;34: 440–540. 10.1016/j.molcel.2009.04.017 19481524 PMC2712825

[22] van den Elzen AM, Henri J, Lazar N, Gas ME, Durand D, Lacroute F, et al Dissection of Dom34-Hbs1 reveals independent functions in two RNA quality control pathways. Nat Struct Mol Biol. 2010;17: 1446–1452. 10.1038/nsmb.1963 21102444

[23] Finck M, Bergmann N, Jansson B, Ernst JF. Defective threonine-linked glycosylation of human insulin-like growth factor in mutants of the yeast *Saccharomyces cerevisiae*. Glycobiology. 1996;6: 313–320. 10.1093/glycob/6.3.313 8724139

[24] Lussier M, Gentzsch M, Sdicu AM, Bussey H, Tanner W. Protein *O*-glycosylation in yeast. The *PMT2* gene specifies a second protein *O*-mannosyltransferase that functions in addition to the *PMT1*-encoded activity. J Biol Chem. 1995;270: 2770–2775. 10.1074/jbc.270.6.2770 7852348

[25] González A, Jiménez A, Vázquez D, Davies JE, Schindler D. Studies on the mode of action of hygromycin B, an inhibitor of translocation in eukaryotes. Biochim Biophys Acta. 1978;521: 459–469. 367435 10.1016/0005-2787(78)90287-3

[26] Dean N. Yeast glycosylation mutants are sensitive to aminoglycosides. Proc Natl Acad Sci USA. 1995;92: 1287–1291. 10.1073/pnas.92.5.1287 7877969 PMC42504

[27] Lay J, Henry LK, Clifford J, Koltin Y, Bulawa CE, Becker JM. Altered expression of selectable marker *URA3* in gene-disrupted *Candida albicans* strains complicates interpretation of virulence studies. Infect Immun. 1998;66: 5301–5306. 9784536 10.1128/iai.66.11.5301-5306.1998 PMC108662

[28] Girrbach V, Strahl S. Members of the evolutionary conserved *PMT* family of protein *O-*mannosyltransferases form distinct protein complexes among themselves. J Biol Chem. 2003;278: 12554–12562. 10.1074/jbc.M212582200 12551906

[29] Tuch BB, Mitrovich QM, Homann OR, Hernday AD, Monighetti CK, De La Vega FM, et al The transcriptomes of two heritable cell types illuminate the circuit governing their differentiation. PLoS Genet. 2010;6: e1001070 10.1371/journal.pgen.1001070 20808890 PMC2924316

[30] Bruno VM, Wang Z, Marjani SL, Euskirchen GM, Martin J, Sherlock G, Snyder M. Comprehensive annotation of the transcriptome of the human fungal pathogen *Candida albicans* using RNA-seq. Genome Res. 2010;20: 1451–1458. 10.1101/gr.109553.110 20810668 PMC2945194

[31] Lilliesfors HW. On the Kolmogorov-Smirnov test for normality with mean and variance unknown. J Am Stat Assoc. 1967;62: 399–402.

[32] Steuten B, Wagner R. A conformational switch is responsible for the reversal of the 6S RNA-dependent RNA polymerase inhibition in *Escherichia coli*. Biol Chem. 2012;393: 1513–1522. 10.1515/hsz-2012-0237 23667906

[33] Koonin EV, Bork P, Sander C. A novel RNA-binding motif in omnipotent suppressors of translation termination, ribosomal proteins and a ribosome modification enzyme? Nucleic Acids Res. 1994;22: 2166–2167. 10.1093/nar/22.11.2166 7518079 PMC308137

[34] Wolfe KH, Shields DC. Molecular evidence for an ancient duplication of the entire yeast genome. Nature. 1997;387: 708–713. 10.1038/42711 9192896

[35] Verma-Gaur J, Traven A. Post-transcriptional gene regulation in the biology and virulence of *Candida albicans*. Cell Microbiol. 2016;18: 800–806. 10.1111/cmi.12593 26999710 PMC5074327

[36] Kadosh D. Control of *Candida albicans* morphology and pathogenicity by post-transcriptional mechanisms. Cell Mol Life Sci., in press. 10.1007/s00018-016-2294-y 27312239 PMC5582595

[37] Sherman F, Fink G, Hicks J. Methods in Yeast Genetics. 1986 Cold Spring Harbor, NY; Cold Spring Harbor Laboratory Press.

[38] Wach A, Brachat A, Pöhlmann R, Philippsen P. New heterologous modules for classical or PCR-based gene disruptions in *Saccharomyces cerevisiae*. Yeast. 1994;10: 1793–1808. 7747518 10.1002/yea.320101310

[39] Morschhäuser J, Michel S, Staib P. Sequential gene disruption in *Candida albicans* by FLP-mediated site-specific recombination. Mol Microbiol. 1999;32: 547–556. 10320577 10.1046/j.1365-2958.1999.01393.x

[40] Timpel C., Strahl-Bolsinger S, Ziegelbauer K, Ernst JF. Multiple functions of Pmt1p-mediated protein *O*-mannosylation in the fungal pathogen *Candida albicans*. J Biol Chem. 1998;273: 20837–20846. 9694829 10.1074/jbc.273.33.20837

[41] Fonzi W, Irwin Y. Isogenic strain construction and gene mapping in *Candida albicans*. Genetics. 1993;134: 717–728. 8349105 10.1093/genetics/134.3.717 PMC1205510

[42] Setiadi ER, Doedt T, Cottier F, Noffz C, Ernst JF. Transcriptional response of *Candida albicans* to hypoxia: linkage of oxygen sensing and Efg1p-regulatory networks. J Mol Biol. 2006;361: 399–411. 10.1016/j.jmb.2006.06.040 16854431

[43] Köhler JR, Fink GR. *Candida albicans* strains heterozygous and homozygous for mutations in mitogen-activated protein kinase signaling components have defects in hyphal development. Proc Natl Acad Sci USA. 1996;93: 13223–13228. 8917572 10.1073/pnas.93.23.13223 PMC24074

[44] Brown JL, Kossaczka Z, Jiang B, Bussey H. A mutational analysis of killer toxin resistance in *Saccharomyces cerevisiae* identifies new genes involved in cell wall (1—>6)-beta-glucan synthesis. Genetics. 1993;133: 837–849. 8462845 10.1093/genetics/133.4.837 PMC1205404

[45] Nasmyth KA, Tatchell K. The structure of transposable yeast mating type loci. Cell. 1980;19: 753–764. 10.1016/S0092-8674(80)80051-1 6244896

[46] Strahl-Bolsinger S, Immervoll T, Deutzmann R, Tanner W. *PMT1*, the gene for a key enzyme of protein *O*-glycosylation in *Saccharomyces cerevisiae*. Proc Natl Acad Sci USA. 1993;90: 8164–8168. 10.1073/pnas.90.17.8164 8367478 PMC47309

[47] Foreman PK, Davis RW. Cloning vectors for the synthesis of epitope-tagged, truncated and chimeric proteins in *Saccharomyces cerevisiae*. Gene. 1994;144: 63–68. 10.1016/0378-1119(94)90204-6 7517907

[48] Umeyama T, Nagai Y, Niimi M, Uehara Y. Construction of FLAG tagging vectors for *Candida albicans*. Yeast. 2002;19: 611–618. 10.1002/yea.863 11967831

[49] Sonneborn A, Tebarth B, Ernst JF. Control of white-opaque phenotypic switching in *Candida albicans* by the Efg1p morphogenetic regulator. Infect Immun. 1999;67: 4655–4660. 10456912 10.1128/iai.67.9.4655-4660.1999 PMC96790

[50] Kapitan M, Eichhof I, Lagadec Q, Ernst JF. Click beetle luciferases as dual reporters of gene expression in *Candida albicans*. Microbiology. 2016;162: 1310–1320. 10.1099/mic.0.000329 27339610

[51] Gildehaus N, Neusser T, Wurm R, Wagner R. Studies on the function of the riboregulator 6S RNA from *E*. *coli*: RNA polymerase binding, inhibition of *in vitro* transcription and synthesis of RNA-directed de novo transcripts. Nucleic Acids Res. 2007;35: 1885–1896. 10.1093/nar/gkm085 17332013 PMC1874619

[52] Göringer HU, Bertram S, Wagner R. The effect of tRNA binding on the structure of 5 S RNA in *Escherichia coli*. A chemical modification study. J Biol Chem. 1984;259: 491–496. 6200475

[53] Garre E, Romero-Santacreu L, De Clercq N, Blasco-Angulo N, Sunnerhagen P, Alepuz P. Yeast mRNA cap-binding protein Cbc1/Sto1 is necessary for the rapid reprogramming of translation after hyperosmotic shock. Mol Biol Cell. 2012;23: 137–150. 10.1091/mbc.E11-05-0419 22072789 PMC3248893

