# Supplementary material for: Dom34 Links Translation to Protein O-mannosylation
Source: PLoS Genet. 2016 Oct 21;12(10):e1006395. doi: 10.1371/journal.pgen.1006395 (PMC5074521; doi:10.1371/journal.pgen.1006395)
Supplement: S1 Fig — Dom34/Pelota sequences of S. cerevisiae (ScDom34), C. albicans (CaDom34), D. melanogaster (DmPelota) and S. pombe (SpDom34) are aligned. The positions of domains 1–3 is indicated by the arrows and the circled numbers. The region suggested as a RNA binding sequence in domain 3 is underlined. The conserved glutamate in domain 1, presumed to act in RNase activity, is indicated by the thick arrow. (PDF) [file pgen.1006395.s001.pdf]

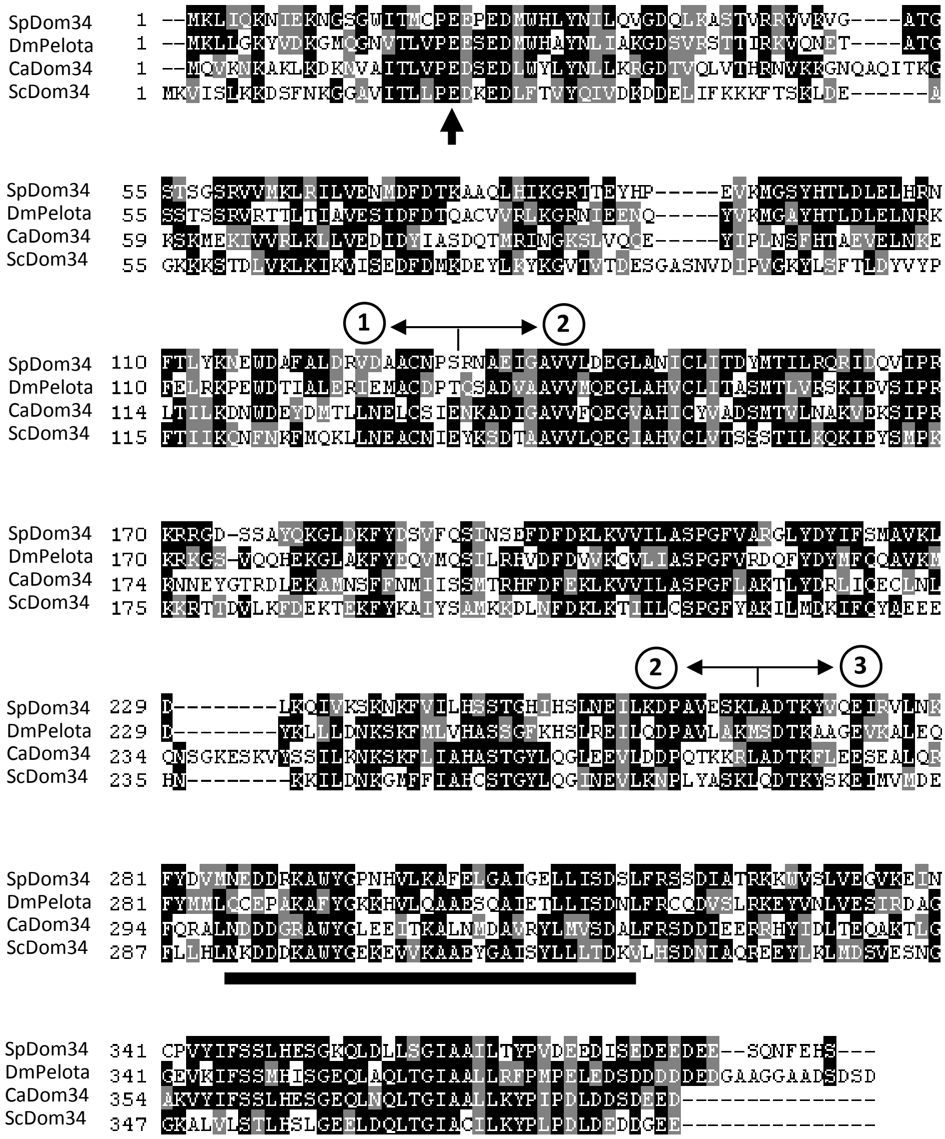

S1 Fig. Structure of Dom34 proteins. Dom34/Pelota sequences of *S. cerevisiae* (ScDom34), *C. albicans* (CaDom34), *D. melanogaster* (DmPelota) and *S. pombe* (SpDom34) are aligned. The positions of domains 1-3 is indicated by the arrows and the circled numbers. The region suggested as a RNA binding sequence in domain 3 isunderlined. The conserved glutamate in domain 1, presumed to act in RNase activity is indicated by the thick arrow.
