# Supplementary material for: Dom34 Links Translation to Protein O-mannosylation
Source: PLoS Genet. 2016 Oct 21;12(10):e1006395. doi: 10.1371/journal.pgen.1006395 (PMC5074521; doi:10.1371/journal.pgen.1006395)
Supplement: S2 Fig — (A) Scheme of wild-type DOM34 locus, after disruption by the FRT-URA3—FLP-FRT cassette and after removal of the URA3-FLP sequences (top-to-bottom). (B) Southern blotting of genomic DNA in transformants. Total DNA was digested with ClaI and SalI and blots were probed using a DOM34 segment indicated by asterisks in (A). The fragment for the wild-type alleles is visible in lane 1, while the subsequent lanes demonstrate the course of disruption in derivative strains and the complete disruption of the two DOM34 alleles in strain SK47 (lane 5). (PDF) [file pgen.1006395.s002.pdf]

A.

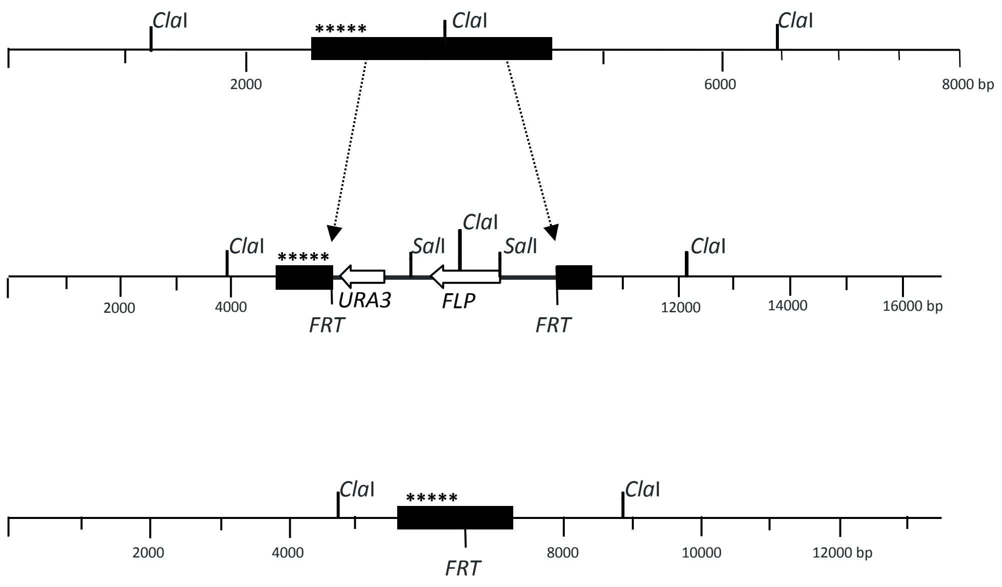

B.

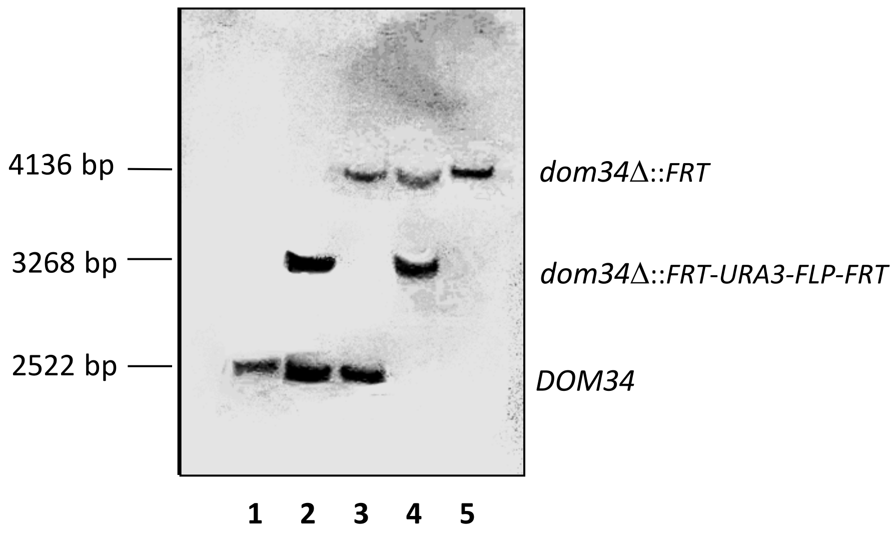

S2 Fig. Disruption of *DOM34* locus. (A) Scheme of wild-type *DOM34* locus, after disruption by the *FRT-URA3—FLP-FRT* cassette and after removal of the *URA3-FLP* sequences (top-to-bottom). **(B)** Southern blotting of genomic DNA in transformants. Total DNA was digested with *Cla*I and *Sal*I and blots were probed using a *DOM34* segment indicated by asterisks in (A). The fragment for the wild-type alleles is visible in lane 1, while the subsequent lanes demonstrate the course of disruption in derivative strains and the complete disruption of the two *DOM34* alleles in strain SK47 (lane 5).
