# Supplementary material for: Dom34 Links Translation to Protein O-mannosylation
Source: PLoS Genet. 2016 Oct 21;12(10):e1006395. doi: 10.1371/journal.pgen.1006395 (PMC5074521; doi:10.1371/journal.pgen.1006395)
Supplement: S3 Fig — Total RNA of strains CAF2-1 (+/+), SPCa2 (pmt1/pmt1), SPCa4 (pmt2/PMT2), SPCa6 (pmt4/pmt4), SPCa10 (pmt5/pmt5) and SPCa8 (pmt6/pmt6) was isolated and the relative DOM34 transcript level (RTL) was determined by qPCR using the ACT1 transcript as the reference. Values obtained for two biological replicates are shown as black and white bars. (PDF) [file pgen.1006395.s003.pdf]

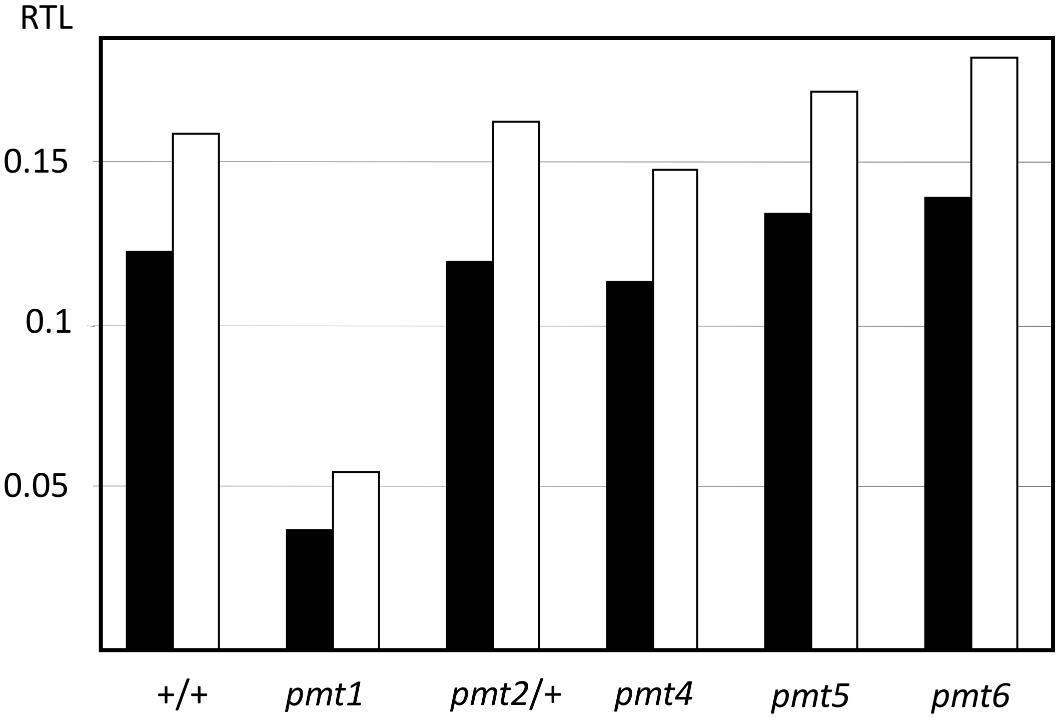

S3 Fig. *DOM34* transcript levels in *pmt* strains. Total RNA of strains CAF2-1 (+/+), SPCa2 (*pmt1*/*pmt1*), SPCa4 (*pmt2*/*PMT2*), SPCa6 (*pmt4*/*pmt4*), SPCa10 (*pmt5*/*pmt5*) and SPCa8 (*pmt6*/*pmt6*) was isolated and the relative *DOM34* transcript level (RTL) was determined by qPCR using the *ACT1* transcript as the reference. Values obtained for two biological replicates are shown as black and white bars.
