# Supplementary material for: Dom34 Links Translation to Protein O-mannosylation
Source: PLoS Genet. 2016 Oct 21;12(10):e1006395. doi: 10.1371/journal.pgen.1006395 (PMC5074521; doi:10.1371/journal.pgen.1006395)
Supplement: S4 Fig — (A) Influence of DOM34 overexpression was tested using strains CAF2-1 (+/+), SPCa2 (pmt1/pmt1), CAP1-3121[pSP38] (pmt1/pmt1[empty vector] and strain CAP1-3121[pSK2] (pmt1/pmt1[DOM34]). (B) Influence of dom34 mutation was tested using strains CAF2-1 (+/+), SPCa2 (pmt1/pmt1), JH24-4 (pmt1/pmt1 dom34/dom34) and JH47-1 (dom34/dom34). Total RNA of all strains was isolated and relative transcript levels of the indicated PMT genes were determined by qPCR using the ACT1 transcript as the reference. Values obtained for two independent biological replicates are shown as black and white bars. (PDF) [file pgen.1006395.s004.pdf]

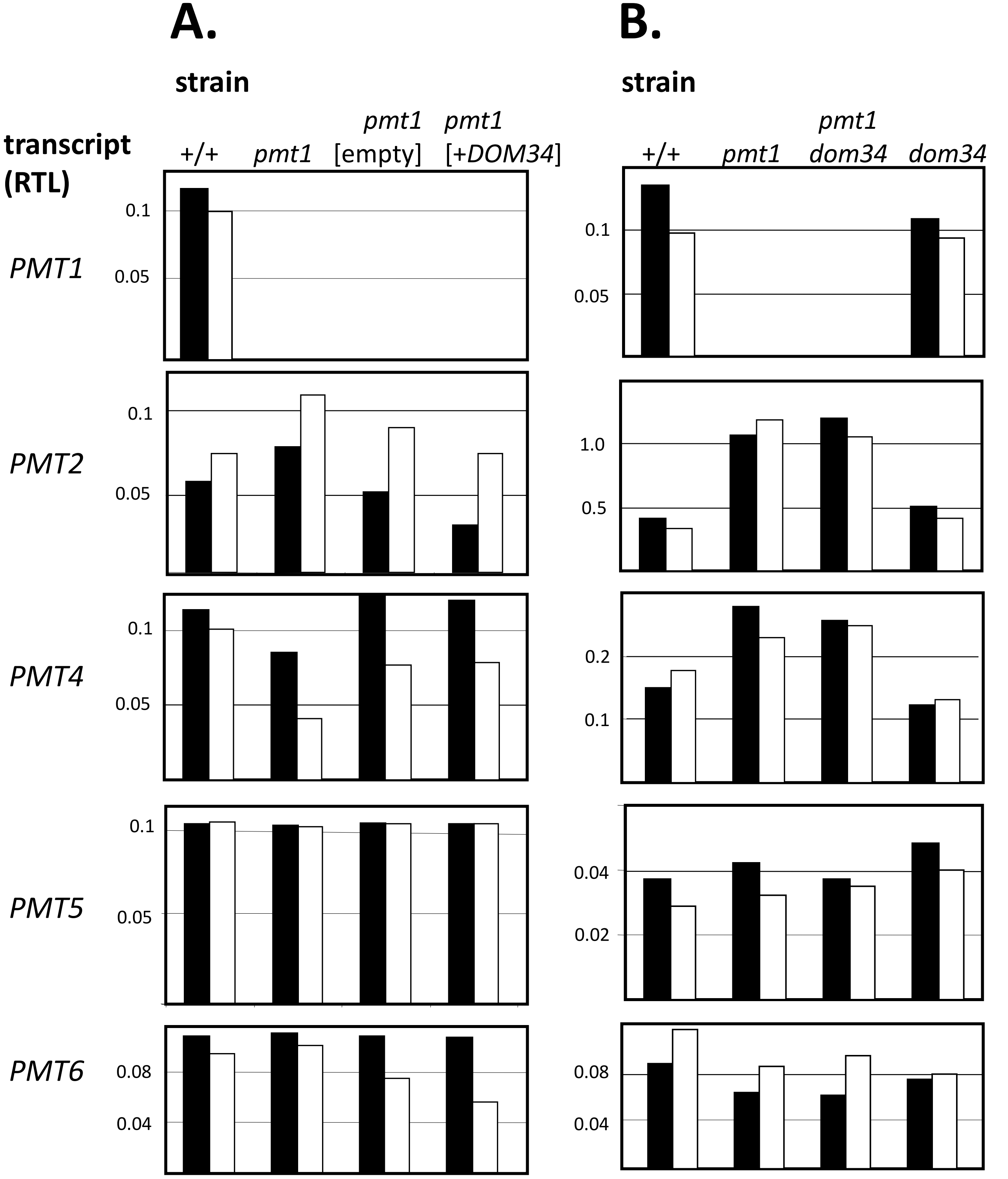

S4 Fig. Influence of *DOM34* expression on *PMT* transcript levels. (A) Influence of *DOM34* overexpression was tested using strains CAF2-1 (+/+), SPCa2 (*pmt1/pmt1*), CAP1-3121[pSP38] (*pmt1/pmt1*[empty vector] and strain CAP1-3121[pSK2] (*pmt1/pmt1*[*DOM34*]). **(B)** Influence of *dom34* mutation was tested using strains CAF2-1 (+/+), SPCa2 (*pmt1/pmt1*), JH24-4 (*pmt1/pmt1 dom34/dom34*) and JH47-1 (*dom34/dom34*). Total RNA of all strains was isolated and relative transcript levels of the indicated *PMT* genes were determined by qPCR using the *ACT1* transcript as the reference. Values obtained for two independent biological replicates are shown as black and white bars.
