# Supplementary material for: Dom34 Links Translation to Protein O-mannosylation
Source: PLoS Genet. 2016 Oct 21;12(10):e1006395. doi: 10.1371/journal.pgen.1006395 (PMC5074521; doi:10.1371/journal.pgen.1006395)
Supplement: S5 Fig — Cells of strains JHCa1-1 (DOM34/DOM34HA) were fractionated by differential centrifugation. The periplasmic space (PE), crude extract (CE), pellet after centrifugation at 10,000 x g (P10; ER fraction), pellet after centrifugation of P10 supernatant at 100,000 x g (P100; Golgi fraction) and the corresponding supernatant (S100; cytoplasmic fraction) were obtained. Aliquots of each fraction were examined by SDS-PAGE (10% acrylamide) followed by immunoblotting using rat anti-HA antibody. The arrow indicates the migration of HA-tagged Dom34. As a control, an immunoblot of a crude extract of strain CIS23 (PMT1/PMT1HA), producing HA-tagged Pmt1, is shown. (PDF) [file pgen.1006395.s005.pdf]

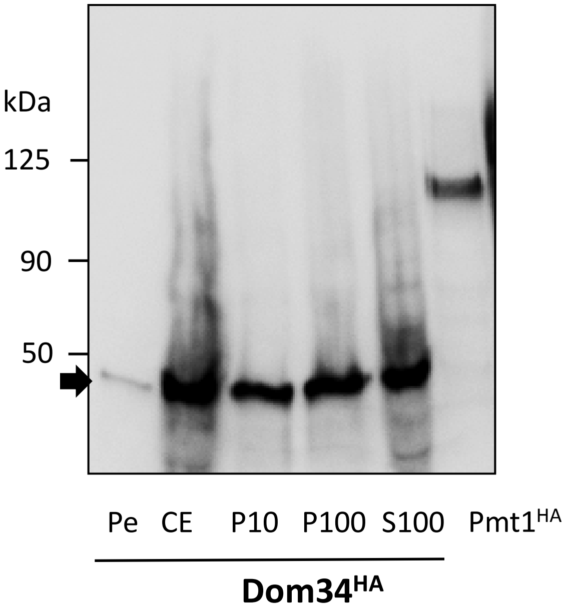

S5 Fig. Immunoblot of HA-tagged Dom34. Cells of strains JHCa1-1 (*Dom34/DOM34*^{HA}) were fractionated by differential centrifugation. The periplasmic space (PE), crude extract (CE), pellet after centrifugation at 10,000 *x g* (P10; ER fraction), pellet after centrifugation of P10 supernatant at 100,000 *x g* (P100; Golgi fraction) and the corresponding supernatant (S100; cytoplasmic fraction) were obtained. Aliquots of each fraction were examined by SDS-PAGE (10 % acrylamide) followed by immunoblotting using rat anti-HA antibody. The arrow indicates the migration of HA-tagged Dom34. As a control, an immunoblot of a crude extract of strain CIS23 (*PMT1/PMT1*^{HA}), producing HA-tagged Pmt1, is shown.
