# Supplementary material for: Dom34 Links Translation to Protein O-mannosylation
Source: PLoS Genet. 2016 Oct 21;12(10):e1006395. doi: 10.1371/journal.pgen.1006395 (PMC5074521; doi:10.1371/journal.pgen.1006395)
Supplement: S6 Fig — (A) The indicated transcripts were detected by qPCR after adding a known amount of an in vitro generated transcript of CaCBGluc as calibrator. Each bar represents the normalized mean PMT4 or PMT6 transcript level of two independent experiments including the standard error of the mean. (B) Incidence of “CAAC” and “ACCA” motifs in 5’-UTRs of ACT1 and PMT-genes. The 5’-UTR sequences of ACT1 (reference gene) and all C. albicans PMT-genes were analyzed for occurrence of the identified “CAAC” and “ACCA” motif (Fig 3B). Information about transcript start sites were taken from Tuch et al. (2010) and Bruno et al. (2010) [29,30]; the transcript with the longer 5’-UTR was chosen and respective sequences were obtained from CGD (http://www.candidagenome.org) assembly 21. Occurrence of the “CAAC” (underlined) and “ACCA” (line on top) motifs are highlighted in red at the indicated positions. The “CAAC” motif was identified in 5’-UTRs of all PMT-genes, but not in the 5’-UTR sequence of the reference gene ACT1. (PDF) [file pgen.1006395.s006.pdf]

A.

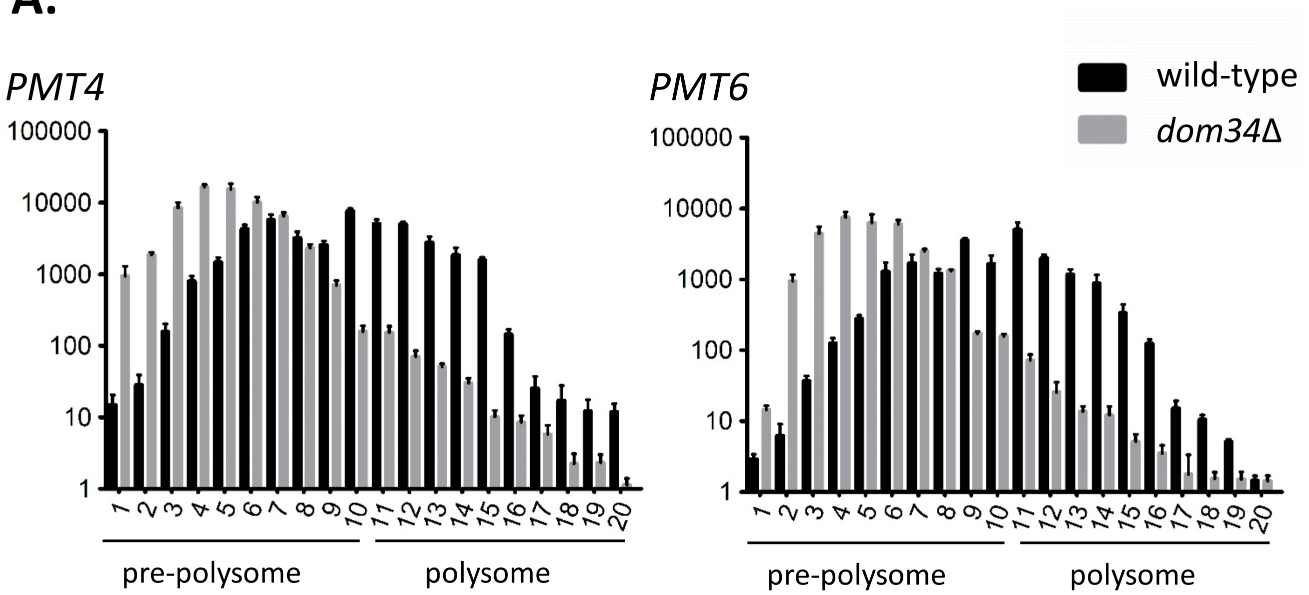
PMT4
PMT6
wild-type
dom34Δ
100000
10000
1000
100
10
1
1 2 3 4 5 6 7 8 9 10 11 12 13 14 15 16 17 18 19 20
pre-polysome
polysome

B.

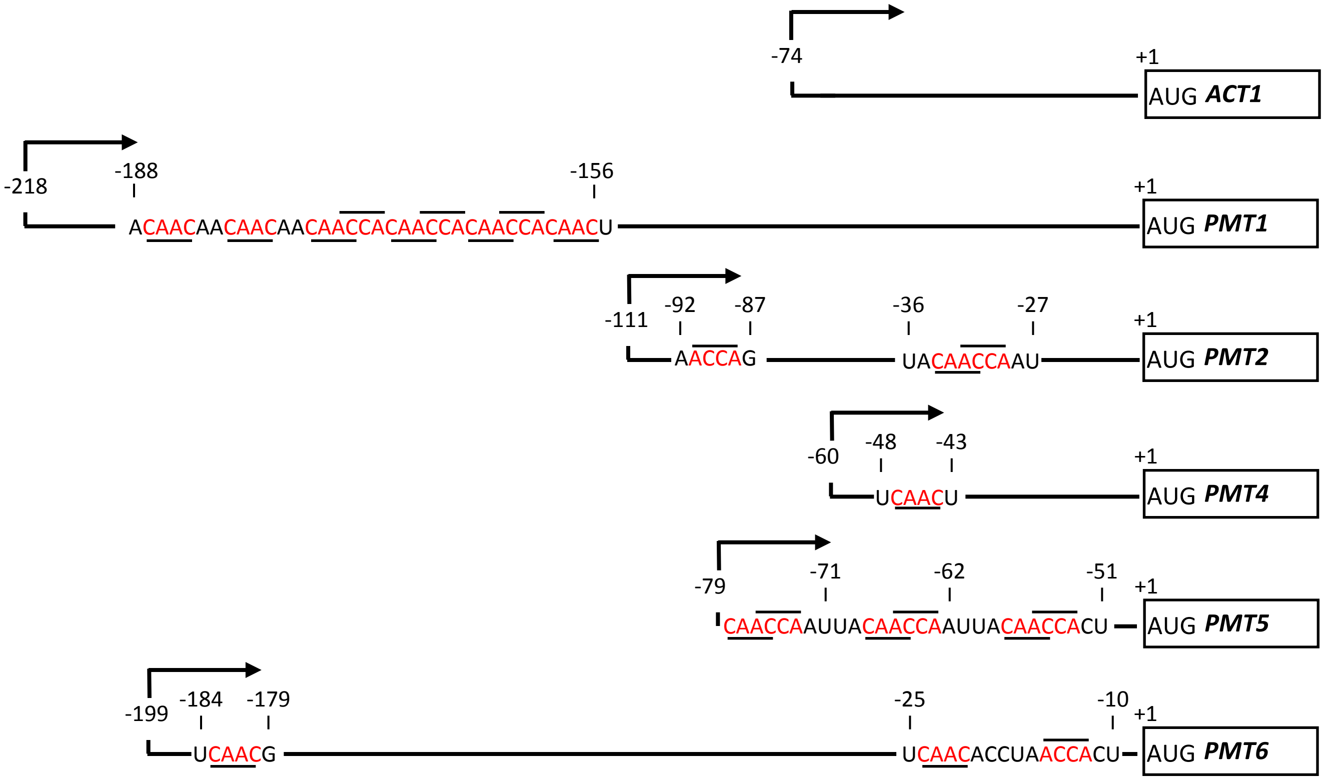
-74
+1
AUG ACT1
-218
-188
-156
ACAACAACAACAACAACCACAACCACAACCACAACU
AUG PMT1
-111
-92
-87
-36
-27
AACCAG
UACAACCAAU
AUG PMT2
-60
-48
-43
UCAACU
AUG PMT4
-79
-71
-62
-51
CAACCAAUUACAACCAAUUACAACCACU
AUG PMT5
-199
-184
-179
-25
-10
UCAACG
UCAACACCUAACCACU
AUG PMT6

S6 Fig. Occurrence of *PMT4* and *PMT6* transcripts in polysome-gradient fractions. (A) The indicated transcripts were detected by qPCR after adding a known amount of an *in vitro* generated transcript of CaCBGluc as calibrator. Each bar represents the normalized mean *PMT4* or *PMT6* transcript level of two independent experiments including the standard error of the mean. **(B)** Incidence of "CAAC" and "ACCA" motifs in 5'-UTRs of *ACT1* and *PMT*-genes. The 5'-UTR sequences of *ACT1* (reference gene) and all *C. albicans PMT*-genes were analyzed for occurrence of the identified "CAAC" and "ACCA" motif (Fig. 3B). Information about transcript start sites were taken from Tuch *et al*. [29] and Bruno *et al*. [30]; the transcript with the longer 5'-UTR was chosen and respective sequences were obtained from CGD (http://www.candidagenome.org) assembly 21. Occurrence of the "CAAC" (underlined) and "ACCA" (line on top) motifs are highlighted in red at the indicated positions. The "CAAC" motif was identified in 5'-UTRs of all *PMT*-genes, but not in the 5'-UTR sequence of the reference gene *ACT1*.
