# Supplementary material for: Dom34 Links Translation to Protein O-mannosylation
Source: PLoS Genet. 2016 Oct 21;12(10):e1006395. doi: 10.1371/journal.pgen.1006395 (PMC5074521; doi:10.1371/journal.pgen.1006395)
Supplement: S7 Fig — E. coli Rosetta transformants carrying pET22b-Dom34+ were induced by IPTG and cell pellets were disrupted by ultrasonication. Soluble proteins in the cell extract were separated by affinity chromatography on Ni-NTA agarose, which was washed in buffer using 5 mM imidazole and eluted using 50 and 250 mM imidazole. Aliquots of fractions were separated by SDS-PAGE (4–20% acrylamide) and gels were stained using Coomassie Brilliant Blue R-250. Lanes show protein standards (S), cell pellet (Pe), flow-through (F), wash fractions (W1,2) and elution fractions (E1-5). The arrow marks the position of Dom34-His6. (PDF) [file pgen.1006395.s007.pdf]

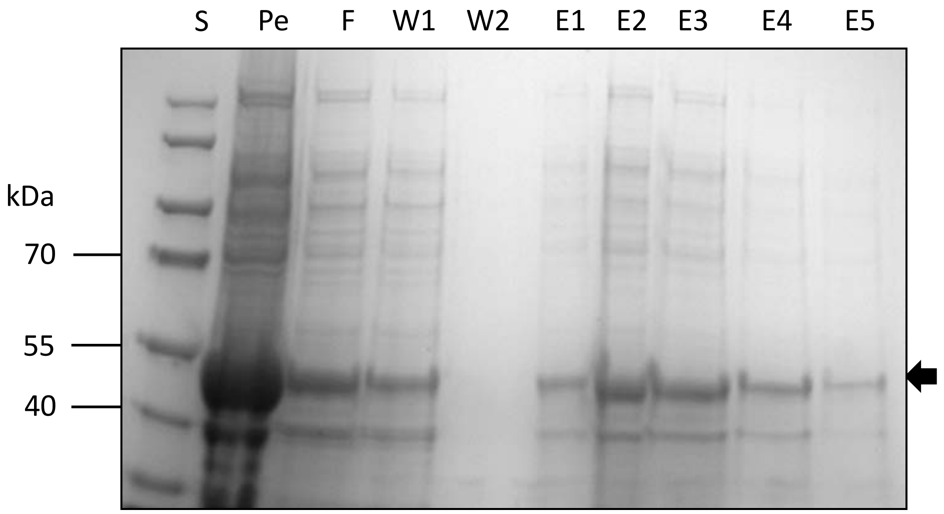

S7 Fig. Purification of Dom34-His$_6$. *E. coli* Rosetta transformants carrying pET22b-Dom34+ were induced by IPTG and cell pellets were disrupted by ultrasonication. Soluble proteins in the cell extract were separated by affinity chromatography on Ni-NTA agarose, which was washed in buffer using 5 mM imidazole and eluted using 50 and 250 mM imidazole. Aliquots of fractions were separated by SDS-PAGE (4-20 % acrylamide) and gels were stained using Coomassie Brilliant Blue R-250. Lanes show protein standards (S), cell pellet (Pe), flow-through (F), wash fractions (W1,2) and elution fractions (E1-5). The arrow marks the position of Dom34-His$_6$.
