# Supplementary material for: Dom34 Links Translation to Protein O-mannosylation
Source: PLoS Genet. 2016 Oct 21;12(10):e1006395. doi: 10.1371/journal.pgen.1006395 (PMC5074521; doi:10.1371/journal.pgen.1006395)
Supplement: S8 Fig — Radioactive 3‘ end-labeled 6S RNA was incubated with increasing amounts of Dom34; after complex formation samples were analysed by 6% native PAGE. As indicated Dom34 was present in final concentrations of 0.1/0.15/0.25/0.3/0.6/1 and 2.5 μM. (PDF) [file pgen.1006395.s008.pdf]

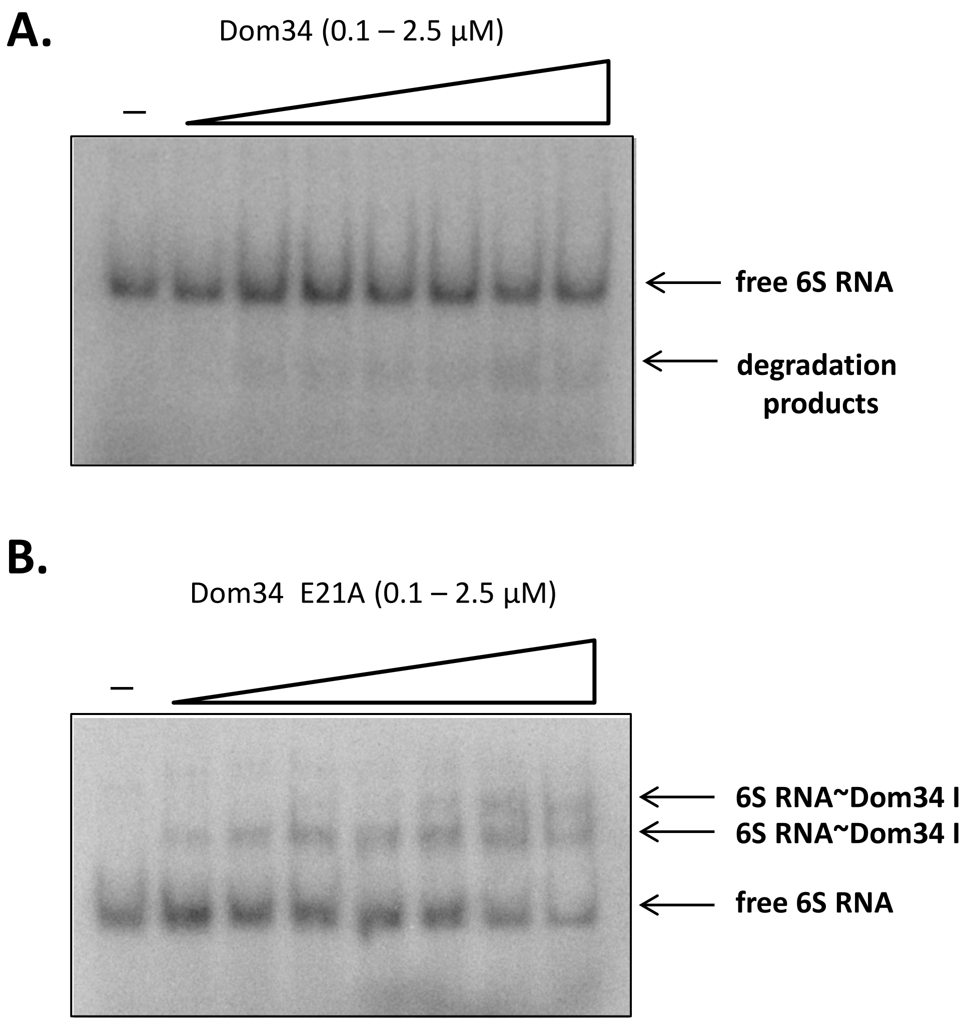

S8 Fig. Weak binding of native Dom34 (A) or the Dom34 E21A variant (B) to 6S RNA from *E. coli*. Radioactive 3′ end-labeled 6S RNA was incubated with increasing amounts of Dom34; after complex formation samples were analysed by 6% native PAGE. As indicated Dom34 was present in final concentrations of 0.1/0.15/0.25/0.3/0.6/1 and 2.5 µM.
