# Supplementary material for: Dom34 Links Translation to Protein O-mannosylation
Source: PLoS Genet. 2016 Oct 21;12(10):e1006395. doi: 10.1371/journal.pgen.1006395 (PMC5074521; doi:10.1371/journal.pgen.1006395)
Supplement: S9 Fig — (A) UTR RNA sequence generated by in vitro transcription of plasmid pRG01 (linearized by BglII) using T7 RNA polymerase. Two G residues are added to 218 nt UTR sequence at its 5′-end by T7 RNA polymerase, while AGATC at the 3′-end reflects the BglII sequence. The transcript start site at -218 was determined by Tuch et al. [29], while an additional start site at -190 was reported by Bruno et al. [30] (underlined U in sequence). The CA/AC-rich region (Dom34 binding site) is marked in red font. (B) Predicted folding structure of PMT1 UTR. The RNAfold program (http://rna.tbi.univie.ac.at/cgi-bin/RNAfold.cgi) was used for prediction and results were depicted as a centroid structure drawing encoding base-pair probabilities (colour code showing probabilities of base-pairing or single strandedness in predicted paired and unpaired regions, respectively). Numbered black arrows indicated predicted events upon Dom34 binding: (1) Binding of Dom34 to single-stranded region containing CA/AC-repeats, (2) opening of paired region by binding of Dom34, (3) opening of paired region and cleavage by Dom34, (4) cleavage by Dom34. (C) RNAse cleavage experiments supporting the predicted actions of Dom34. 3′-[32P] end-labelled UTR RNA (50 cps) was incubated with RNases in the absence or presence of Dom34 for 1 min at 37°C; Dom34 was preincubated 10 min with the UTR before RNAse addition. Products were separated by 12% denaturing PAGE. Nucleotide positions are numbered from the UTR 5′-end as in B. UTR, no RNAse (lane 1); UTR with 1 U RNAse T1 cleaving at G residues indicated in the left margin (lane 2); UTR with 0.5 U RNAse U2 specific for RNA single strands (lane 3); UTR with 1 μM Dom34 (lane 4); UTR sequence ladder generated by partial hydrolysis with NaOH (lane 5); UTR with 0.25/0.5/1 U RNAse U2 (lanes 6–8); UTR incubated with 0.5 U RNAse U2 and 0.5/1/2.5 μM Dom34 (lanes 9–11). Note a prominent Dom34-mediated cleavage of the UTR around position 100. RNase U2 cleavage in position 30–60 (CA/ [file pgen.1006395.s009.pdf]

A.

1 20 40 60

*GG*CAAGAUUCUUUUUCAAGAUUUUUCAUUUUUACAACAACAACAACAACCACCACAACCACA

80 100 120

ACUAUAAACAAUUGAUAUAGCAUUAUCUUUUAAGAUUAUUGAAUAAAGGUCAAUUUAUAGCG

140 160 180

CCCCCCCUCACUCCACCUCCUACCGUCUUUCUUUCACGCAACUAAAACAAAUUUUUAUAUUU

200 218

UUAUUUUUAUUUUUAUUUUAGUUUCCCAUUCAAU*AGAUC*-3´

B.

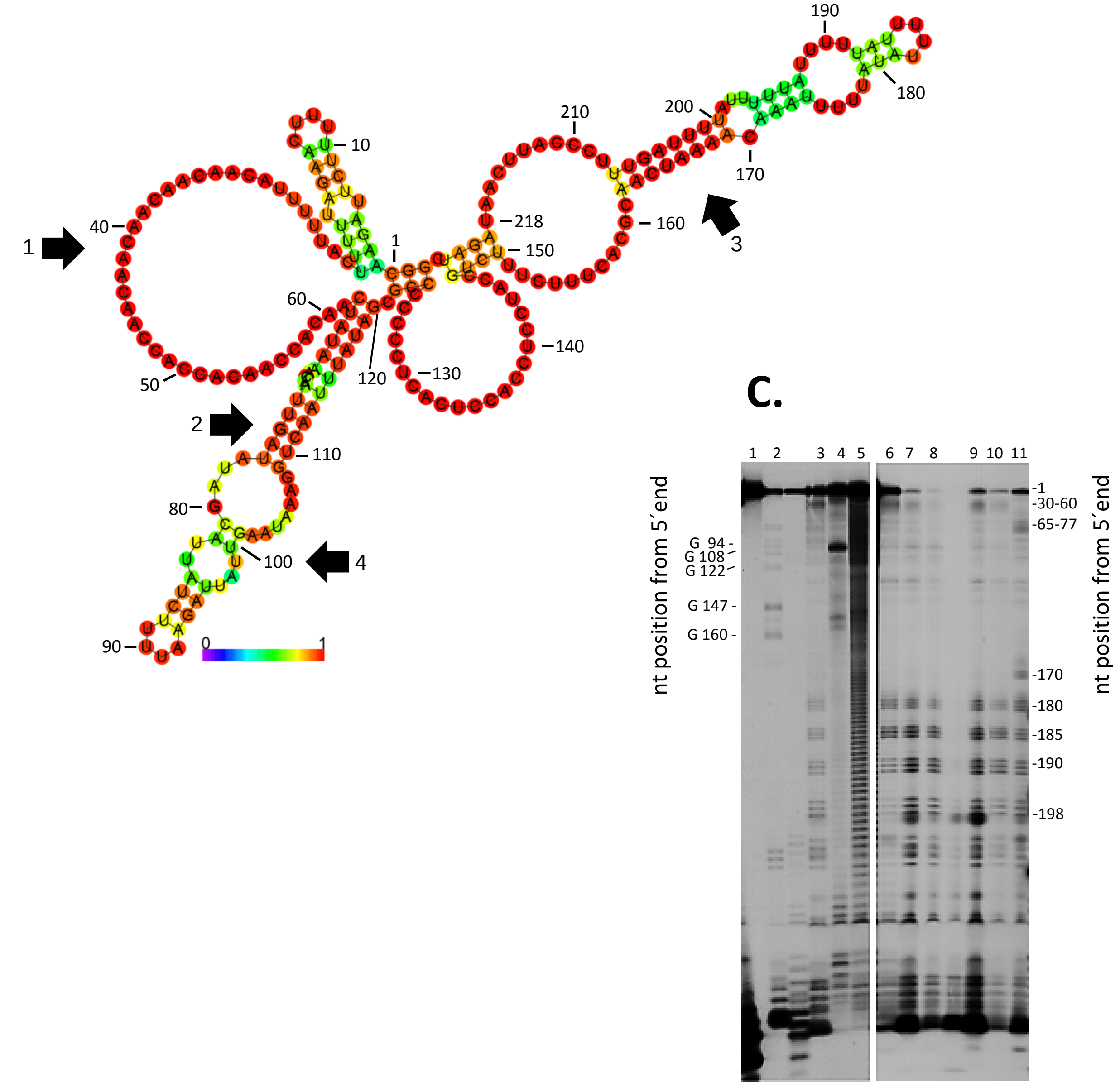

S9 Fig. Sequence and structure of the *PMT1* 5´-UTR. (A) UTR RNA sequence generated by *in vitro* transcription of plasmid pRG01 (linearized by *Bgl*II) using T7 RNA polymerase. Two G residues are added to 218 nt UTR sequence at its 5´-end by T7 RNA polymerase, while AGATC at the 3´-end reflects the *Bgl*II sequence. The transcript start site at -218 was determined by Tuch *et al.* [29], while an additional start site at -190 was reported by Bruno *et al.* [30] (underlined U insequence). The CA-enriched region (Dom34 binding site) is marked in red font. (**B)** Predicted folding structure of *PMT1* UTR. The RNAfold program (http://rna.tbi.univie.ac.at/cgi-bin/RNAfold.cgi) was used for prediction and results were depicted as a centroid structure drawing encoding base-pair probabilities (colour code showing probabilities of base-pairing or single strandedness in predicted paired and unpaired regions, respectively). Numbered black arrows indicated predicted events upon Dom34 binding: (1) Binding of Dom34 to single-stranded region containing CA-repeats, (2) opening of paired region by binding of Dom34, (3) opening of paired region and cleavage by Dom34 , (4) cleavage by Dom34. (**C)** RNase cleavage experiments supporting the predicted actions of Dom34. 3´-[^{32}P] end-labelled UTR RNA (50 cps) was incubated with RNases in the absence or presence of Dom34 for 1 min at 37 °C; Dom34 was preincubated 10 min with the UTR before RNase addition. Products were separated by 12 % denaturing PAGE. Nucleotide positions are numbered from the UTR 5´-end as in B. UTR, no RNase (lane 1); UTR with 1 U RNase T1 cleaving at G residues indicated in the left margin (lane 2); UTR with 0.5 U RNase U2 specific for RNA single strands (lane 3); UTR with 1 µM Dom34 (lane 4); UTR sequence ladder generated by partial hydrolysis with NaOH (lane 5); UTR with 0.25/0.5/1 U RNase U2 (lanes 6-8); UTR incubated with 0.5 U RNase U2 and 0.5/1/2.5 µM Dom34 (lanes 9-11). Note a prominent Dom34-mediated cleavage of the UTR around position 100. RNase U2 cleavage in position 30-60 (CA-repeat region) confirms the predicted single-strandedness of the UTR in this position (lanes 7-9); Dom34 protects this region (lanes 10, 11), confirming its predicted binding at this site. Region 180-190 appears permanently single-stranded, while the predicted double-stranded regions 65-77 and around 170 become single-stranded upon Dom34 binding (lanes 9-11).
