# Supplementary material for: Dom34 Links Translation to Protein O-mannosylation
Source: PLoS Genet. 2016 Oct 21;12(10):e1006395. doi: 10.1371/journal.pgen.1006395 (PMC5074521; doi:10.1371/journal.pgen.1006395)
Supplement: S1 Table — (PDF) [file pgen.1006395.s010.pdf]

S1 Table. Strains.

	Genotype or description	Source
<u>*C. albicans*</u> <u>strains</u>		
CAI4	*ura3Δ::imm434/ura3Δ::imm434*	[1]
CAF2-1	*ura3Δ::imm434/URA3*	[1]
RM1000	*ura3::imm434/ura3::imm434 iro1/iro1::imm434 his1::hisG/his1::hisG*	[2]
SK47	as CAI4 but *dom34Δ::FRT/dom34Δ::FRT*	this work
JH47-1 /-2	as CAI4 but *dom34Δ ::FRT/dom34Δ ::FRT ura3Δ ::imm434/URA3*	this work
SK24	as CAI4 but *pmt1Δ::hisG/pmt1Δ::hisG dom34Δ::FRT/dom34Δ::FRT*	this work
JH24-4	*pmt1Δ::hisG/pmt1Δ::hisG dom34Δ::FRT/ dom34Δ::FRT ura3Δ ::imm434/URA3*	this work
JH5-3-1	as SK47 but *pmt5Δ-hisG-URA3-hisG/pmt5Δ-hisG*	this work
P15-274	as SK47 but *pmt1Δ::hisG/pmt1Δ::hisG pmt5Δ-URA3/pmt5Δ-hisG*	this work
P15-274-1	as SK47 but *pmt1Δ::hisG/pmt1Δ::hisG pmt5Δ-hisG/pmt5Δ-hisG*	this work
CIS23	as CAI4 but *PMT1/PMT1*^{HA}*-SAT1*	this work
CAP1-3121	as CAI4, but *pmt1Δ::hisG /pmt1Δ::hisG*	[3]
SPCa2	as CAI4, but *pmt1Δ::hisG /pmt1Δ::hisG ura3Δ::imm434/URA3*	[4]
SPCa10	as CAI4, but *pmt5Δ::hisG/pmt5Δ::hisG ura3Δ::imm434/URA3*	[4]
CPP1121	as CAI4, but *pmt1Δ::hisG/pmt1Δ::hisG pmt6Δ-hisG/pmt6Δ-hisG*	[4]
SPCa8	as CAI4, but *pmt6Δ::hisG/pmt6Δ::hisG ura3Δ::imm434/URA3*	[4]
SPCa4	as CAI4, but *PMT2/pmt2Δ::hisG ura3Δ::imm434/URA3*	[4]
SPCa6	as CAI4, but *pmt4Δ::hisG/pmt4Δ::hisG ura3Δ::imm434/URA3*	[4]
JH-Ca1-1 /-2	as CAI4, but *DOM34/DOM34*^{HA}*-URA3*	this work
<u>*S. cerevisiae*</u> <u>strains</u>		
YE449	*MATα leu2 ura3-52 prb1-112 pep4-3 cir*^{0}	[5]
YE449 *pmt1*	as YE449, but *pmt1::URA3*	this work
YE449 *pmt2*	as YE449, but *pmt2::LEU2*	this work
YE449 *pmt1 pmt2*	as YE449, but *pmt1::URA3 pmt2::LEU2*	this work

YE449 *dom34*	as YE449, but *dom34Δ::kanMX4*	this work
W21, W32	as YE449, but *pmt1Δ::URA3 dom34Δ::kanMX4*	this work
W12, W14	as YE449, but *pmt1Δ::URA3 yil001wΔ::kanMX4*	this work
M577	as YE449, but mutant isolate after EMS mutagenesis	[5]
M577 *pmt1*	as M577, but *pmt1::URA3*	this work
M577 *pmt2*	as M577, but *pmt2::LEU2*	this work
M577 *pmt1 pmt2*	as M577, but *pmt1::URA3 pmt2::LEU2*	this work
RC130	*MATa/MATα his1/his1* [*KIL-K1*] (K^+ R^+)	R. Chan, unpublished

References

1. Fonzi W, Irwin Y. Isogenic strain construction and gene mapping in *Candida albicans*. Genetics. 1993;134: 717-728.

2. Negredo A, Monteoliva L, Gil C, Pla J, Nombela C. Cloning, analysis and one-step disruption of the *ARG5,6* gene of *Candida albicans*. Microbiology. 1997;143: 297-302.

3. Timpel C., Strahl-Bolsinger S, Ziegelbauer K, Ernst JF. Multiple functions of Pmt1p-mediated protein *O*-mannosylation in the fungal pathogen *Candida albicans*. J Biol Chem. 1998;273: 20837-20846.

4. Prill SK-H, Klinkert B, Timpel C, Gale CA, Schröppel K, Ernst JF. *PMT* family of *Candida albicans*: five protein mannosyltransferase isoforms affect growth, morphogenesis and antifungal resistance. Mol Microbiol. 2005;55: 546-560.

5. Finck M, Bergmann N, Jansson B, Ernst JF. Defective threonine-linked glycosylation of human insulin-like growth factor in mutants of the yeast *Saccharomyces cerevisiae.* Glycobiology. 1996;6: 313-320.
