# Supplementary material for: Dom34 Links Translation to Protein O-mannosylation
Source: PLoS Genet. 2016 Oct 21;12(10):e1006395. doi: 10.1371/journal.pgen.1006395 (PMC5074521; doi:10.1371/journal.pgen.1006395)
Supplement: S2 Table — (PDF) [file pgen.1006395.s011.pdf]

S2 Table. Oligonucleotides.

name	sequence (5′-3′)
***S. cerevisiae* genes**	
ScDOM34 disrupt for	ATGAAGGTTATTAGTCTGAAAAAGGATTCTTTTAACAAAGCAGCT GAAGCTTCGTACGC
ScDOM34 disrupt rev	CTACTCCTCACCATCGTCTTCATCAAGATCGGGGAGCGGGGCATA GGCCACTAGTGGATCTG
ScYIL001w disrupt for	ATGGCAGATAAATTAATGGACAAAAATTTTGAAGAACTGTCAGCT GAAGCTTCGTACGC
ScYIL001w disrupt rev	TTATACATCAAGTTCCAGATCAGCTAGGATATTATCTAACGCATAG GCCACTAGTGGATCTG
ScDOM34N	TTTGAATTCTTATGAAGGTTATTAGTCTG
ScDOM34C	TTTCTGCAGGCTACTCCTCACCATCGTC
ScYIL001wN	TTTGAATTCTTATGGCAGATAAATTAATGG
ScYIL001wC	TTTCTGCAGGTTATACATCAAGTTCCAG
YEp13-Bamflank-A	ACTATCGACTACGCG
YEp13-Bamflank-B	CCGGATCCAGCAACCGCACCTGTG
***C. albicans* genes**	
FDP34	ATTCCGCGGTCCCAGAACCAATCAAGATTAATTACG
RPD34	ATTGCGGCCGCGTGGTGATGCTATAAGAATCTTTTCTG
FDD34	ATTCTCGAGGGAACTACATTATTTTTTATACGAGTT
RDD34	ATTGGGCCCTTCATTATGTTTTGCAGCAATTATGAT
Dom34-NdeI-FW	GGAATTCCATATGGCTGGCACAGTTCCTGGCCA
Dom34-XhoI-RV-Stopp	CGCTGAGGTCTTCTTCATCACTGTC
Dom34-Leu(mut)for	CTAAATTTTTGGAGGAGTCGGAGGCATTACAGCGC
Dom34-Leu(mut)rev	GCGCTGTAATGCCTCCGACTCCTCCAAAAATTTAG
Dom34Mut21fw	GCAATAACCTTGGTACCCGCTGATTCCGAGGATTTATGG
Dom34Mut21rev	CCATAAATCCTCGGAATCAGCGGGTACCAAGGTTATTGC
Dom34Mut317fw	AAAGGCATTAGCTATGGATGCAG
Dom34Mut317rev	CTGCATCCATAGCTAATGCCTTT
p1-DOM-FLAG	TTACTGCAGATGCAAGTTAAAAACAAAGC
p2-DOM-FLAG	TTAGCATGCCAAGTCTTCTTCATCACTG
Dom34-HA-for	GAATCTGGTGAACAATTGAATCAGTTGACGGGTATTGC TGCTTTATTGAAATATCCGATACCAGATCTTGATGACAGTGATGAA GAAGACGGTGGTGGTCGGATCCCCGGGTTAATT AA
Dom34-HA-rev	GGTTTAATACAGGACTATTTAATTCACGCCAGAAAGGG TAGAGTCTGGTATTTAGATAGGTATACACAACAAAAAC TCGTATAAAAAATAATACTCTAGAAGGACCACCTTTGA TTG
CaPmt1del-for	GAAAAGCAACAACAAGAACAACAACAAGAACAAGAACAGGTTGA AGATGAATCAGTGCATCAAGTTCAACAAGGTGGTGGTCGGATCCC CGGGTTAATTAA

CaPmt1del-rev	CAAATATGTAAAATGTGATGTGTAATATATGTTCATATTTCTCTTTCTGCAAAAGATAATTTCTTCTACTAAGTCTAGAAGGACCACCTTTGATTG
Pmt1 5´UTR for	CAAGATTCTTTTTCAAGATTTTTC
Pmt1 5´UTR rev BglII long	AAAAAGATCTATTGAATGGGAAACTAAAATAAAAATAA
CBG-Stu for	CCTACAAGATCTATGGTTAAGAGAGAAAAAAACG
CBG-Bam rev	GGATCCTTAACCACCAGCTTTTTC
ACT1(RT)-rev	TGGACAAATGGTTGGTCAAG
ACT1(RT)-for	TTGGATTCTGGTGATGGTGT
PMT1-RT-for	GCTGCTGAACCTGTTGAAGA
PMT1-RT-rev	CATCAGCAACTTGTGGGTCT
PMT2-for-RTa	CCATGATGGCTACTAACAATG
PMT2-rev-RTa	CCCATCCACACATTCTAATACC
PMT4-RT-for	TTGGAAACATCATTGGGTTC
PMT4-RT-rev	TTGATCTTGCTCTGTCGCTT
PMT5-RT-for	CCCTTATGCATCTCCTCCAT
PMT5-RT-rev	CACCAAATATGGCCAGGAAT
PMT6-RT-for	ATTTGTTGGTTGTTGGCATC
PMT6-RT-rev	TGATTGACTGTTTGCTGGGT
DOM34-RT-for	GTTGAATGATGACGACGGAA
DOM34-RT-rev	CGTCGCTTCTAAACAAAGCA
PMT1 5’-UTR RNA	ACAACAACAACAACAACCACCACAACCACAACUAUAAACAAU
